# A systematic review of the accuracy of digital surgical guides for dental implantation

**DOI:** 10.1186/s40729-023-00507-w

**Published:** 2023-10-25

**Authors:** Yiting Shi, JunKai Wang, Chao Ma, Jiayi Shen, Xian Dong, Dan Lin

**Affiliations:** Shanghai University of Medicine and Health Sciences, Shanghai, 201318 People’s Republic of China

**Keywords:** Surgical guides, Guided implant surgery, Digital dentistry, Surgical accuracy, Deviation, Systematic review

## Abstract

**Purpose:**

This review aimed to reveal the influence of implant guides on surgical accuracy with regard to supporting types, manufacturing methods and design (including fixation screws and sleeves).

**Methods:**

A literature search related to accuracy of surgical guides for dental implantation was performed in Web of Science and PubMed. Studies with in vivo or in vitro deviation data published in recent 5 years (2018–2022) were included and assessed by Newcastle–Ottawa Scale with regard to risk of bias and reliability degree of clinical studies. Accuracy-related deviation data were summarized as forest plots and normal distributions.

**Results:**

Forty-one articles were included with high degree of credibility. Data showed that implant surgery accuracy can be achieved with mean distance deviation < 2 mm (most < 1 mm) and angular deviation < 8° (most < 5°).

**Conclusions:**

Bilateral tooth-supported guides exhibited highest in vitro accuracy and similar in vivo accuracy to unilateral tooth-supported guides; mucosa-supported guides exhibit lowest in vivo accuracy, while its in vitro data showed low credibility due to mechanical complexity of living mucosa tissue. Milling exhibited higher in vivo accuracy of guides than 3d-printing, though further data support was needed. Design of fixation screws and sleeves of implant guides affected the surgical accuracy and might remain a research focus in near future. However, lack of universal evaluation standards for implantation accuracy remained a major problem in this field. The influence of implant guides on surgical accuracy revealed in this review might shed light on future development of dental implantology.

**Supplementary Information:**

The online version contains supplementary material available at 10.1186/s40729-023-00507-w.

## Introduction

Global population aging results in increasing demand for dental implant surgery, emphasizing the necessity of improving the surgical accuracy, which directly increases the success rate and reduces surgical trauma [1]. In recent years, digital technology that realizes the visualization of planting schemes significantly raises the surgical accuracy of dental implantation [2]. Digital surgical guides, as the information carrier of implant direction, position, angle, can effectively enhance the surgical accuracy, reduce surgical time and complications [3]. The surgical accuracy of dental implantation is influenced by data acquisition method, manufacturing procedure, supporting types, fixation screws and sleeve design of the surgical guide.

**Fig. 1:**
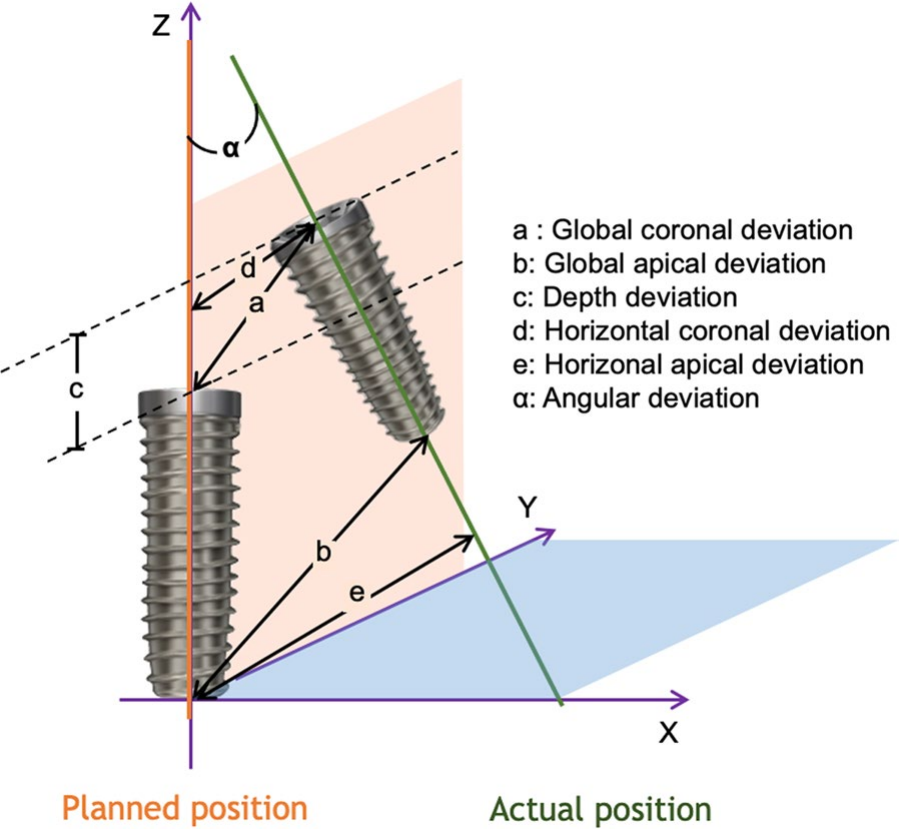
The indicators of implant surgical accuracy commonly applied in existing literatures

Accuracy of implanting guide comprises trueness and precision (ISO 5725–1:1994). Trueness refers to the deviation between postoperative placement and preoperative plan of the implant; precision refers to the deviation of repetitive test results. Generally, accuracy discussed in clinical studies refers to trueness, while in vitro studies (e.g., implant on plaster models) may involve both trueness and precision. The accuracy compared and discussed in this review mainly refers to trueness. Despite the lack of a universal evaluation standard for implantation surgical accuracy, common indicators including coronal deviation (mm), apical deviation (mm), depth deviation/vertical deviation (mm), angular deviation (°) are applied in existing literatures and discussed in this review (Fig. 1).

Preoperative data acquisition, including intraoral (collected via intraoral scanning or extraoral scanning of impressions) and CBCT data, is the prerequisite for implant guide design [4]. Existing studies indicated a higher accuracy of intraoral scanners (IOSs) than extraoral scanning of impressions [5], and IOSs are the developing trend in the future and are highly accepted among the patients due to its flexibility and simplicity [6]. Accuracy of commercial IOSs ranges from 20 to100 μm for dentition, and 50–250 μm for edentulous jaws [7, 8]. By contrast, CBCT exhibits lower accuracy: within an applicable range of radiation dose (20–100 μSv, generally considered as a balance of safety and accuracy), the voxel size of CBCT-constructed 3D models ranges from 0.1 to 0.5 mm^3^, and the accuracy ranges from 200 to 1000 μm [9]. Considering the accuracy range of CBCT, it is generally recommended to maintain a safety margin of 2 mm from adjacent anatomical structures in clinical practice [10]. As the accuracy of dentition model obtained by CBCT is relatively low to meet the requirements of surgical guide design, integration of CBCT-constructed model and scanned dentition model is commonly applied [4]. As data acquisition accuracy will be projected onto design of surgical guides, and current evaluation of implant surgery accuracy is mainly based on CBCT data acquisition, comparability of the accuracy indicator values in different literatures should be reviewed dialectically.

Two common narrative aspects of existing reviews on the accuracy of digital surgical guides are comparing static surgical guides to dynamic or augmented reality (AR) navigation systems [11] and analyzing the accuracy of surgical guides for specific conditions. Examples of the later include comparison of the applicability, accuracy and clinical effects of digital and traditional surgical guides in flapless implant surgery reviewed by Emitis Natali Naeini in 2020 [12] demonstrating higher accuracy of digital surgical guides than traditional ones; comparison of the accuracy of digital surgical guides between flap and flapless implant surgery by Karthikeyan Subramani in 2022 [13] indicating that flapless surgery resulted in higher accuracy; analysis of the accuracy of edentulous mucosa-supported surgical guides prepared by Stereolithography by Cheongbeom Seo in 2018 [14] and surgical guides for partially edentulous patients analyzed by Ramadhan Hardani Putra in 2022 [3], both exhibiting no significant differences in any specific influencing factors. In addition, R. Eftekhar Ashtiani in 2021 [15] compares the accuracy among different guiding systems and concludes that both the implant and its design software influenced the accuracy, though no final statement could be made on an optimized system. Fernando Bover-Ramos in 2018 [16] compares the accuracy of surgical guides studied in cadaveric, clinical, and in vitro models and finds that in vitro studies showed higher accuracy than clinical and cadaveric studies, and the accuracy of full-guided surgical guides are higher than half-guided ones. A review published by Firas Al Yafi in 2019 [1] summarizes the operational procedures of digital surgical guides in detail, and though lack of in-depth data analyses, provides a comprehensive list of accuracy-affecting factors. A review by Ali Tahmaseb in 2018 [17], based on published research data from 2012–2015, concludes that the accuracy of surgical guide was within the clinically acceptable range in most cases and was higher in partially edentulous patients than edentulous patients.

Considering the lack of a systematic and comprehensive review of the factors influencing the accuracy of surgical guides, this review analyzed and discussed the accuracies of all static digital surgical guides in aspects including guide supporting types, manufacturing methods and design of implant guides (including fixation screws and sleeves), covering different influencing factors to provide a comprehensive guidance for implant design in future. This review aimed to reveal the influence of implant guides on surgical accuracy, and to provide reference for future development of digital dental implantology. It was hypothesized that these aspects influence the accuracies of static digital surgical guides, and the hypothesis was verified by collecting and categorizing the numerical data of surgical accuracy indicator reported in literature of the last five years.

## Materials and methods

This review has been registered in PROSPERO, with the registration ID of 416029.

The search was performed using keywords based on the PICO approach. The PICO was formulated as follows: Participants (P) = patients of dental implantation; Intervention (I) = implants placed using digital surgical guides; Comparison or control (C) = different supporting types, design of fixation screws, design of sleeve, manufacturing methods of the surgical guides; Outcome measures (O) = coronal deviation (mm), apical deviation (mm), depth deviation (mm), angular deviation (°).

Based on the above PICO analysis, this review applied the keywords: computer-aided implant surgery (CAIS); static surgical guide; accuracy; deviation; dental implants) and MeSH terms (Surgery, Computer-Assisted) AND (Dental Implants). Advanced searching strategies were established based on the above keywords to perform an extensive search of the literature for papers related to accuracy of digital surgical guides for dental implantation on the databases of Web of Science (WOS) and PubMed as follows:

WOS: TS = (oral OR dental) AND TS = (surgical guide) AND TS = (accuracy OR precision OR rightness) AND TS = (implant OR implantation).

PubMed: (oral OR dental) AND (surgical guide) AND (accuracy OR precision OR rightness) AND (implant OR implantation).

Literatures were screened using predetermined inclusion and exclusion criteria as follows.

Inclusion criteria:

1. Types of literature were limited to research articles, case reports and clinical trials that were peer-reviewed and published in WOS or PubMed cited scientific journals.
2. Titles and abstracts of the articles were related to the accuracy of digital surgical guides for dental implantation.
3. At least one of the following in vivo or in vitro deviation data must be involved: coronal deviation (mm), apical deviation (mm), depth deviation (mm), angular deviation (°).
4. Written in English.
5. The year of publication was restricted in recent five years (2018–2022).

Exclusion criteria:

1. Reviews, meeting abstracts, grey literature or non-peer-reviewed literature were excluded.
2. Written in languages other than English.
3. Published before 2018.

To minimize the potential for reviewer bias, two reviewers (CM and JS) independently conducted literature searches and performed the study selection. Both reviewers strictly followed the inclusion and exclusion criteria, and any disagreement was resolved by discussion.

Data were extracted by one reviewer (JS) and examined by another reviewer (CM). The following data were directly collected from the included articles: literature information (authors, year, and title), research type (clinical/cadaver/in vitro), number of patients/cadavers/models, number of implants, surgical information (full-/half-guided, planning software, implant site, jaw position, bone quality and implant length and diameter), types of surgical guide (bilateral tooth-supported, unilateral tooth-supported, and mucosa-supported guides), guide fabrication method (3D printing/milling), number of fixation pins, deviation data including global/horizontal coronal deviation (mm), global/horizontal apical deviation (mm), angular deviation (°) and vertical deviation (mm). The form of deviation data included mean ± SD and/or median (min, max).

To assess the risk of bias and degree of reliability, clinical studies were scored based on the Newcastle–Ottawa Scale (NOS) adapted by Chambrone et al. [18] including evaluation of four subcategories: sample selection of study groups, comparability, outcome and statistical analysis. Specific scoring items are listed in Additional file 1: Methods. A maximum of 13 points could be obtained for each study, with a score of 10–13 indicating high study quality, a score of 7–9 indicating moderate study quality, and a score of less than 7 indicating low study quality.

## Results

**Fig. 2:**
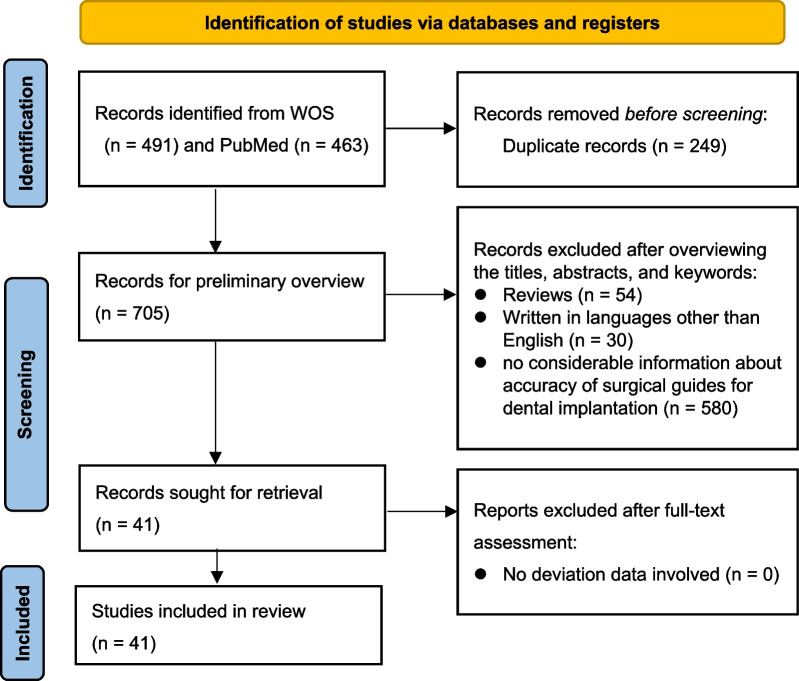
Search flowchart according to PRISMA guidelines [19]. (*n* = number of records)

Following the PRISMA guideline (Fig. 2), the search strategy reported 954 records, among which 249 duplicate records were firstly removed. After overviewing the titles, abstracts and keywords, the investigators excluded 54 reviews, 30 articles written in languages other than English, and 580 records with no considerable information about accuracy of surgical guides for dental implantation. The remaining 41 records were sought for full-text retrieval and assessment of data availability, and all 41 articles involved available deviation data. The 41 articles were ultimately included in this review, among which 21 were in vitro studies, 19 were in vivo studies, and 1 was a comparison of in vitro and in vivo accuracy. Among the 19 in vivo studies, 2 were performed on cadaver, and 17 were clinical researches. Among 17 clinical research types, 3 were case–control studies, 11 were clinical trials, and 3 were cohort studies. The comparison of in vitro and in vivo accuracy was a case–control study (Table 1).

**Table 1:** Classification of research types

Type of research	Number	Type of clinical study	Number
In vitro	21	–	–
In vivo			
Cadaver	2	–	–
Clinical	17	Case–control study	3
		Clinical trial	11
		Cohort study	3
Comparison of in vitro and in vivo	1	Case–control study	1

NOS analysis was performed in the 20 in vivo studies in this paper, of which 3 were of medium quality and 17 were of high quality (Additional file 1: Fig. S1). Specifically, all studies exhibited high scores in sample selection, comparability and statistics, though the outcomes of patient follow-up adequacy were presented in only 3 studies. The NOS results indicated a high degree of credibility of this review.

A descriptive table of the 41 studies included in this review with deviation data including global/horizontal coronal deviation (mm), global/horizontal apical deviation (mm), angular deviation (°) and vertical deviation (mm) in the form of mean ± SD and/or median (min, max) was provided as Table 2, and the data listed and discussed in following part of this review are based on the corresponding information. The comparison criteria (supporting types, design of fixation screws, design of sleeve, manufacturing methods of the surgical guides) and accuracy-related deviation data of the included literatures were summarized as forest plots (Tables 3, 4, 5, Additional file 1: Tables S1–S4) and normal distributions (Figs. 4, 5).

**Table 2:** Descriptive table of the 41 studies included in this review with deviation data including global/horizontal coronal deviation (mm), global/horizontal apical deviation (mm), angular deviation (°) and vertical deviation (mm) in the form of mean ± SD and/or median (min, max)

Ref. No.	Author (year)	Research type	Clinical research type	Full- or half-guided	No. of patients/models	No. of implants	Implant site	Supported type	Fabrication
1	Xiaoqian Liu (2022) [20]	In vitro	/	/	/	54	/	/	Milling
2	Roberto Pessoa (2022) [21]	In vitro	/	Full	16	48	26, 27, 28	Unilateral tooth-supported	3D printer
		In vitro	/	Full	16	48	26, 27, 28	Unilateral tooth-supported	3D printer
3	Rani D'haese (2022) [22]	In vitro	/	Full	15	15	26	Bilateral tooth-supported	3D printer
4	Yao Sun (2022) [23]	In vitro	/	/	10	20	36, 46	Bilateral tooth-supported	3D printer
		In vitro	/	/	10	20	36, 46	Bilateral tooth-supported	3D printer
5	Jeanette K Li-Rodríguez (2022) [24]	In vitro	/	Full	8	24	15, 36, 46	Bilateral tooth-supported	Milling
		In vitro	/	Full	8	8	21	Bilateral tooth-supported	Milling
6	Nicole Báez-Marrero (2022) [25]	In vitro	/	/	8	15	2, 7, 8	Unilateral tooth-supported	3D printer
7	Kristof Orban (2022) [26]	In vivo	Cohort study	Half	20	20	4, 5 or 6, 7	Unilateral tooth-supported	3D printer
8	Jordi Gargallo-Albiol (2022) [27]	In vivo	Cohort study	Full	30	60	4, 5 or 6, 7	Unilateral tooth-supported	3D printer
9	Wanwanat Singthong (2022) [28]	In vivo	Clinical trial	Full	12	12	4, 5 or 6, 7	Bilateral tooth-supported	3D printer
		In vivo	Clinical trial	Full	12	12	4, 5 or 6, 7	Bilateral tooth-supported	3D printer
10	Yuzhang Feng (2022) [29]	In vivo	Clinical trial	Full	20	20	1, 2	Bilateral tooth-supported	3D printer
11	Fangzhi Lou (2021) [30]	In vivo	Clinical trial	Half	20	36	11, 12, 13, 21, 22, 23	Bilateral tooth-supported	3D printer
		In vivo	Clinical trial	Full	20	33	11, 12, 13, 21, 22	Bilateral tooth-supported	3D printer
12	David Schneider (2021) [31]	In vitro	/	Half	24	72	34, 35, 36	Bilateral tooth-supported	/
		In vitro	/	Full	24	72	34, 35, 36	Bilateral tooth-supported	3D printer
		In vitro	/	Half	24	24	46	Bilateral tooth-supported	/
		In vitro	/	Full	24	24	46	Bilateral tooth-supported	3D printer
13	Young Woo Song (2021) [32]	In vitro	/	Full	20	20	36	Bilateral tooth-supported	Milling
		In vitro	/	Full	20	20	36	Bilateral tooth-supported	3D printer
14	Jaafar Abduo (2021) [33]	In vitro	/	Full	14	14	13	Bilateral tooth-supported	Milling
		In vitro	/	Full	14	14	16	Bilateral tooth-supported	Milling
		In vitro	/	Pilot	14	14	13	Bilateral tooth-supported	3D printer
		In vitro	/	Pilot	14	14	16	Bilateral tooth-supported	3D printer
15	Laura Herschdorfer (2021) [34]	In vitro	/	Full	10	10	46	Bilateral tooth-supported	3D printer
		In vitro	/	Full	10	10	46	Bilateral tooth-supported	3D printer
		In vitro	/	Full	10	10	46	Bilateral tooth-supported	3D printer
16	Chalermchai Ngamprasertkit (2021) [35]	In vivo	Clinical trial	Half	15	15	1, 2, 3, 4, 5	Bilateral tooth-supported	3D printer
		In vivo	Clinical trial	Full	15	15	1, 2, 3, 4, 5	Bilateral tooth-supported	3D printer
17	Johannes Spille (2021) [36]	In vitro	/	Half	6	48	1, 2, 4, 5, 6, 7	Mucosa-supported	/
18	Arndt Guentsch (2021)[37]	In vitro	/	Half (6 mm-sleeve)	20	20	46	Bilateral tooth-supported	3D printer
		In vitro	/	Full (2 mm-sleeve)	20	20	46	Bilateral tooth-supported	3D printer
		In vitro	/	Full (4 mm-sleeve)	20	20	46	Bilateral tooth-supported	3D printer
		In vitro	/	Full (6 mm-sleeve)	20	20	46	Bilateral tooth-supported	3D printer
19	Yen-Ting Han (2021)[38]	In vivo	Clinical trial	Half	30 (Total)	18	/	/	3D printer
		In vivo	Clinical trial	Full	30 (Total)	56	/	/	3D printer
		In vivo	Clinical trial	Full	30 (Total)	23	/	/	3D printer
		In vivo	Clinical trial	Full	30 (Total)	33	/	/	3D printer
		In vivo	Clinical trial	Full	30 (Total)	28	/	Mucosa-supported	3D printer
		In vivo	Clinical trial	Full	30 (Total)	20	/	Bilateral tooth-supported	3D printer
20	Paknisa Sittikornpaiboon (2021) [39]	In vitro	/	/(5 mm-sleeve)	5	10	14, 24	Bilateral tooth-supported	3D printer
		In vitro	/	/(5 mm-sleeve)	5	10	14, 24	Bilateral tooth-supported	3D printer
		In vitro	/	/(4 mm-sleeve)	5	10	14, 24	Bilateral tooth-supported	3D printer
		In vitro	/	/(4 mm-sleeve)	5	10	14, 24	Bilateral tooth-supported	3D printer
		In vitro	/	/(4 mm-sleeve)	5	10	14, 24	Bilateral tooth-supported	3D printer
21	Lirong Huang (2021)[40]	In vivo	Case–control study	Full	20	21	1, 2, 3	Bilateral tooth-supported	/
		In vivo	Case–control study	Full	20	31	1, 2, 3	Bilateral tooth-supported	/
22	Rani D’haese (2021)[41]	In vitro	/	Full	15	90	46, 44, 42, 32, 34, 36	Mucosa-supported	3D printer
		In vitro	/	Full	15	90	46, 44, 42, 32, 34, 36	Mucosa-supported	3D printer
23	Chia-Cheng Lin (2020) [42]	In vitro	/	Full	30	30	36	Unilateral tooth-supported	3D printer
		In vitro	/	Full	30	30	46	Unilateral tooth-supported	3D printer
		In vitro	/	Full	30	30	47	Unilateral tooth-supported	3D printer
24	Yuan Chen (2020) [43]	In vivo	Case–control study	Full	30	37	1, 2, 3	Bilateral tooth-supported	3D printer
		In vivo	Case–control study	Half	33	39	1, 2, 3	Bilateral tooth-supported	3D printer
25	Dong Wu (2020) [44]	In vivo	Case–control study	/	29	57	1, 2, 4, 5, 6, 7	Bilateral tooth-supported	3D printer
26	Kang-jie Cheng (2020) [45]	In vitro	/	Full	1	5	35, 36, 45, 46, 47	Unilateral tooth-supported	3D printer
27	Pantip Henprasert (2020) [46]	In vitro	/	Full	15	15	46	Bilateral tooth-supported	milling
		In vitro	/	Full	15	15	46	Bilateral tooth-supported	3D printer
28	Kristian Kniha (2020) [47]	In vivo	Clinical trial	Full	5	10	1, 2, 3	Bilateral tooth-supported	3D printer
		In vivo	Clinical trial	Full	5	10	4, 5, 6, 7	Bilateral tooth-supported	3D printer
		In vivo	Clinical trial	Full	5	10	1, 2, 3	Mucosa-supported	3D printer
		In vivo	Clinical trial	Full	5	10	4, 5, 6, 7	Mucosa-supported	3D printer
29	R. Vinci (2020) [48]	In vivo	Cohort study	Full	12	51	1, 2, 3, 4, 5, 6, 7	Mucosa-supported	Milling
		In vivo	Cohort study	Full	11	49	1, 2, 3, 4, 5, 6, 7	Mucosa-supported	Milling
		In vivo	Cohort study	Full	14	46	1, 2, 3	Mucosa-supported	Milling
		In vivo	Cohort study	Full	14	54	4, 5, 6, 7	Mucosa-supported	Milling
30	Nopparat Suksod (2020) [49]	In vivo	Clinical trial	Half	20	20	1, 2	Bilateral tooth-supported	3D printer
31	Márton Kivovics (2020) [50]	In vivo	Clinical trial	Half	6	18	/	Mucosa-supported	3D printer
		In vivo	Clinical trial	Half	7	22	/	Mucosa-supported	3D printer
32	Palita Smitkarn (2019) [51]	In vivo	Clinical trial	Full	52	60	1, 2, 4, 5, 6, 7	Unilateral tooth-supported	3D printer
33	Karim El Kholy (2019) [52]	In vitro	/	Full	40	240	15, 12, 21, 23, 25, 26	Unilateral tooth-supported	3D printer
		In vitro	/	Full	15	45	15, 12, 23	Bilateral tooth-supported	3D printer
		In vitro	/	Full	15	45	15, 12, 23	Bilateral tooth-supported	3D printer
		In vitro	/	Full	15	45	15, 12, 23	Bilateral tooth-supported	3D printer
34	Henrik Skjerven (2019) [53]	In vivo	Clinical trial	Full	20	27	1, 2, 3, 4, 5, 6, 7	Bilateral tooth-supported	3D printer
35	Rai-Jei Chang (2018) [54]	In vitro	/	Half	17	20	36, 37	Unilateral tooth-supported	/
		In vivo	Case–control study	Half	17	20	36, 37	Unilateral tooth-supported	/
36	Zhaozhao Chen (2018) [55]	In vivo	Clinical trial	/	4	12	11, 12, 21, 22	Bilateral tooth-supported	/
37	Jan Brandt (2018) [56]	In vitro	/	Full	/	30	46	Bilateral tooth-supported	/
38	Philipp Kauffmann (2018) [57]	In vitro	/	/	/	20	44, 45, 46, 47	Mucosa-supported	/
		In vitro	/	/	/	20	44, 45, 46, 47	Mucosa-supported	/
39	Yiqin Fang (2018) [58]	In vivo	Clinical trial	/	32	40	11, 22	Bilateral tooth-supported	3D printer
40	Boyoung Ma (2018) [59]	In vivo	Clinical trial	/	17	28	36, 15	Bilateral tooth-supported	3D printer
		In vivo	Clinical trial	/	17	28	36, 15	Bilateral tooth-supported	3D printer
41	Björn Gjelvold (2018) [60]	In vitro	/	Full	10	10	44	Bilateral tooth-supported	3D printer
		In vitro	/	Full	10	10	44	Bilateral tooth-supported	3D printer

Ref. No.	Author (year)	Data extraction (mean ± SD, median, (min, max))						No. of fixation screws
		Global coronal deviation (mm)	Horizontal coronal deviation (mm)	Global apical deviation (mm)	Horizontal apical deviation (mm)	Angular deviation (°)	Vertical deviation (mm)	
1	Xiaoqian Liu (2022) [20]	0.16 ± 0.06, /, (0.03, 0.29)	/	/	/	0.61 ± 0.40, /, (0.01, 1.86)	0.11 ± 0.07, /, (0.00, 0.25)	/
2	Roberto Pessoa (2022) [21]	0.88 ± 0.36, /, /	0.67 ± 0.22, /, /	1.60 ± 0.69, /, /	1.72 ± 0.70, /, /	4.53 ± 2.04, /, /	–0.16 ± 0.62, /, /	4
		0.88 ± 0.36, /, /	0.55 ± 0.32, /, /	1.44 ± 0.75, /, /	1.63 ± 0.69, /, /	4.28 ± 2.01, /, /	–0.5 ± 0.5, /, /	0
3	Rani D'haese (2022) [22]	0.52 ± 0.25, /, (0.09, 1.07)	/	0.90 ± 0.47, /, (0.14, 1.74)	/	2.63 ± 1.69, /, (0.38, 5.99)	0.32 ± 0.27, /, (0.02, 1.00)	/
4	Yao Sun (2022) [23]	0.35 ± 0.11, /, (0.20, 0.64)	0.21 ± 0.13, /, (0.03, 0.44)	0.75 ± 0.28, /, (0.21, 1.17)	0.48 ± 0.30, /, (0.01, 0.91)	2.74 ± 1.24, /, (0.50, 4.80)	0.11 ± 0.09, /, (0.00, 0.36)	/
		0.41 ± 0.13, /, (0.16, 0.66)	0.28 ± 0.14, /, (0.04, 0.52)	0.91 ± 0.34, /, (0.34, 1.38)	0.60 ± 0.33, /, (0.10, 1.13)	3.22 ± 1.55, /, (1.00, 6.90)	0.11 ± 0.08, /, (0.00. 0.29)	/
5	Jeanette K Li-Rodríguez (2022) [24]	/	0.2 ± 0.126, /, /	/	/	1.1 ± 0.834, /, /	/	/
		/	0.2 ± 0.126, /, /	/	/	1.1 ± 0.834, /, /	/	/
6	Nicole Báez-Marrero (2022) [25]	1.43 ± 0.60, /, /	/	2.19 ± 0.63, /, /	/	6.81 ± 3.10, /, /	/	2
7	Kristof Orban (2022) [26]	1.20 ± 0.46, /, /	1.06 ± 0.52, /, /	1.45 ± 0.79, /, /	1.28 ± 0.83, /, /	4.82 ± 2.07, /, /	0.55 ± 0.28, /, /	/
8	Jordi Gargallo-Albiol (2022) [27]	0.21 ± 0.69, /, /	/	0.67 ± 1.06, /, /	/	5.62 ± 4.09, /, /	/	/
9	Wanwanat Singthong (2022) [28]	/	1.07 ± 0.36, /, (0.47, 1.60)	/	/	3.52 ± 1.64, /, (0.60, 6.10)	− 0.71 ± 0.29, /, (− 0.04, − 1.15)	/
		/	0.97 ± 0.33, /, (0.33. 1.38)	/	/	3.77 ± 2.16, /, (1.05, 7.20)	− 0.84 ± 0.30, /, (− 0.26, − 1.28)	/
10	Yuzhang Feng (2022) [29]	0.99 ± 0.63, /, /	/	1.50 ± 0.75, /, /	/	3.07 ± 2.18, /, /	/	1
11	Fangzhi Lou (2021) [30]	0.69 ± 0.10, 0.675, (0.51, 0.87)	/	0.80 ± 0.08, 0.80, (0.68, 0.95)	/	3.16 ± 0.70, 3.15, (1.43, 4.73)	0.52 ± 0.11, 0.505, (0.33, 0.73)	/
		0.39 ± 0.12, 0.39, (0.15, 0.61)	/	0.28 ± 0.09, 0.29, (0.11, 0.44)	/	2.05 ± 0.45, 2.09, (0.89, 3.00)	0.24 ± 0.06, 0.25, (0.12, 0.34)	/
12	David Schneider (2021) [31]	/	0.70 ± 0.48, 0.56, (0.14, 1.80)	/	0.77 ± 0.53, 0.64, (0.16, 2.04)	1.70 ± 0.67, 1.65, (0.80, 3.20)	0.46 ± 0.33, 0.36, (0.00, 1.26)	
		/	0.18 ± 0.11, 0.15, (0.02, 0.49)	/	0.31 ± 0.17, 0.27, (0.09, 0.83)	1.57 ± 0.84, 1.40, (0.20, 3.30)	0.19 ± 0.13, 0.17, (0.01, 0.44)	
		/	0.49 ± 0.33, 0.43, (0.06, 1.22)	/	0.51 ± 0.33, 0.53, (0.06, 1.24)	1.36 ± 0.78, 1.20, (0.00, 2.80)	0.45 ± 0.46, 0.24, (0.04, 1.70)	
		/	0.24 ± 0.13, 0.21, (0.03, 0.52)	/	0.34 ± 0.20, 0.30, (0.07, 0.93)	1.32 ± 0.88, 1.05, (0.30, 3.40)	0.28 ± 0.19, 0.29, (0.02, 0.78)	
13	Young Woo Song (2021) [32]	/	1.37, 1.01, /, /	/	1.68, 1.41, /, /	3.49, 3.62, /, /	0.95, 0.71, /, /	
		/	0.95, 0.78, /, /	/	1.34, 1.25, /, /	3.04, 2.69, /, /	0.64, 0.44, /, /	
14	Jaafar Abduo (2021) [33]	/	0.46 ± 0.23, /, (0.11, 0.89)	/	0.62 ± 0.42, /, (0.12, 1.46)	1.25 ± 0.84, /, (0.30, 2.66)	0.31 ± 0.26, /, (0.03, 0.84)	
		/	0.39 ± 0.24, /, (0.01, 0.75)	/	0.71 ± 0.41, /, (0.21, 1.53)	1.59 ± 1.13, /, (0.17, 4.20)	0.37 ± 0.30, /, (0.03, 1.08)	
		/	0.53 ± 0.26, /, (0.21, 0.98)	/	1.49 ± 0.54, /, (0.16, 2.27)	6.76 ± 2.49, /, (1.60, 9.65)	0.61 ± 0.35, /, (0.11, 1.18)	
		/	0.34 ± 0.24, /, (0.02, 0.83)	/	0.76 ± 0.52, /, (0.22, 2.16)	4.00 ± 2.62, /, (0.76, 9.07)	0.51 ± 0.47, /, (0.05, 1.77)	
15	Laura Herschdorfer (2021) [34]	0.24 ± 0.19, 0.19, (0.07, 0.71)	/	0.40 ± 0.23, 0.36, (0.08, 0.92)	/	1.44 ± 0.61, 1.30, (0.70, 2.60)	/	
		0.23 ± 0.13, 0.20, (0.08, 0.50)	/	0.37 ± 0.22, 0.34, (0.37, 0.80)	/	1.37 ± 0.71, 1.15, (0.60, 2.40)	/	
		0.22 ± 0.06, 0.23, (0.13, 0.31)	/	0.30 ± 0.08, 0.32, (0.16, 0.39)	/	0.94 ± 0.48, 0.56, (0.00, 1.50)	/	
16	Chalermchai Ngamprasertkit (2021) [35]	0.74 ± 0.36, /, (0.09, 1.38)	0.57 ± 0.39, /, (0.08, 1.38)	1.29 ± 0.61, /, (0.36, 2.32)	1.17 ± 0.68, /, (0.04, 2.31)	3.44 ± 1.61, /, (0.95, 6.68)	(coronal)0.36 ± 0.27, /, (0.01, 0.95) (apical)0.37 ± 0.27, /, (0.03, 1.01)	
		0.48 ± 0.22, /, (0.20, 0.87)	0.39 ± 0.26, /, (0.08, 0.87)	0.71 ± 0.31, /, (0.18, 1.34)	0.64 ± 0.37, /, (0.03, 1.33)	2.03 ± 1.00, /, (0.88, 4.03)	(coronal)0.19 ± 0.14, /, (0.01, 0.51) (apical)0.20 ± 0.13, /, (0.03, 0.51)	
17	Johannes Spille (2021) [36]	/	1.009 ± 0.415, /, /	/	1.068 ± 0.384, /, /	2.67 ± 1.58, /, /	/	
18	Arndt Guentsch (2021)[37]	0.20 ± 0.14, /, /	/	0.19 ± 0.13, /, /	/	2.85 ± 1.47, /, /	/	/
		0.10 ± 0.13, /, /	/	0.12 ± 0.11, /, /	/	1.35 ± 0.52, /, /	/	/
		0.37 ± 0.17, /, /	/	0.37 ± 0.17, /, /	/	1.47 ± 0.62, /, /	/	/
		0.23 ± 0.17, /, /	/	0.23 ± 0.17, /, /	/	1.79 ± 0.57, /, /	/	/
19	Yen-Ting Han (2021)[38]	1.84 ± 0.64, /, (0.92, 3.13)	1.12 ± 0.40, /, (0.41, 1.68)	2.24 ± 0.97, /, (0.65, 3.54)	1.57 ± 0.96, /, (0.34, 3.32)	6.44 ± 3.02, /, (2.09, 14.00)	1.26 ± 0.90, /, (0.03, 2.90)	/
		0.97 ± 0.45, /, (0.00, 2.19)	0.69 ± 0.41, /, (0.00, 1.82)	1.27 ± 0.58, /, (0.00, 3.18)	1.04 ± 0.58, /, (0.00, 2.90)	3.21 ± 1.72, /, (0.01, 7.25)	0.57 ± 0.43, /, (0.00, 1.77)	/
		1.07 ± 0.54, /, /	0.90 ± 0.47, /, /	1.34 ± 0.77, /, /	1.18 ± 0.75, /, /	3.67 ± 2.14, /, /	0.49 ± 0.41, /, /	/
		0.90 ± 0.37, /, /	0.54 ± 0.29, /, /	1.22 ± 0.40, /, /	0.95 ± 0.41, /, /	2.89 ± 1.30, /, /	0.62 ± 0.44, /, /	/
		0.98 ± 0.37, /, /	0.62 ± 0.33, /, /	1.18 ± 0.47, /, /	0.88 ± 0.49, /, /	3.12 ± 1.53, /, /	0.64 ± 0.44, /, /	/
		0.89 ± 0.45, /, /	0.65 ± 0.36, /, /	1.25 ± 0.62, /, /	1.08 ± 0.57, /, /	3.04 ± 1.89, /, /	0.53 ± 0.40, /, /	/
20	Paknisa Sittikornpaiboon (2021) [39]	0.56 ± 0.19, 0.51, (0.32, 0.96)	/	0.83 ± 0.32, 0.75, (0.49, 1.49)	/	2.70 ± 1.37, 2.95, (0.90, 5.10)	/	/
		0.42 ± 0.12, 0.41, (0.25, 0.63)	/	0.76 ± 0.22, 0.73, (0.45, 1.14)	/	2.50 ± 0.89, 2.70, (0.70, 3.60)	/	/
		1.18 ± 0.19, 1.13, (0.86, 1.48)	/	1.70 ± 0.41, 1.63, (1.08, 2.38)	/	4.37 ± 1.34, 4.00, (2.70, 6.50)	/	/
		1.09 ± 0.12, 1.09, (0.90, 1.25)	/	1.95 ± 0.48, 1.98, (0.94, 2.53)	/	5.13 ± 1.86, 5.45, (0.70, 6.90)	/	/
		0.81 ± 0.15, 0.83, (0.47, 1.01)	/	1.73 ± 0.23, 1.72, (1.41, 2.07)	/	5.30 ± 1.04, 5.45, (3.60, 6.50)	/	/
21	Lirong Huang (2021)[40]	0.82 ± 0.32, /, /	/	1.18 ± 0.41, /, /	/	3.24 ± 1.33, /, /	0.64 ± 0.36, /, /	1
		0.89 ± 0.34, /, /	/	1.10 ± 0.38, /, /	/	3.38 ± 1.86, /, /	0.25 ± 0.77, /, /	1
22	Rani D’haese (2021)[41]	0.82 ± 0.43, /, (0.17, 2.08)	/	0.99 ± 0.45, /, (0.12, 2.06)	/	3.25 ± 1.69, /, (0.16, 8.70)	/	4
		0.45 ± 0.31, /, (0.05, 1.62)	/	0.71 ± 0.43, /, (0.15, 2.14)	/	2.39 ± 1.42, /, (0.37, 8.16)	/	4
23	Chia-Cheng Lin (2020) [42]	0.78 ± 0.57, /, (0.06, 2.97)	0.47 ± 0.33, /, (0.04, 1.39)	1.29 ± 0.88, /, (0.07, 3.53)	1.10 ± 0.80, /, (0.05, 3.35)	3.67 ± 2.73, /, (0.16, 11.32)	0.57 ± 0.53, /, (0.05, 2.76)	/
		0.75 ± 0.47, /, (0.22, 2.37)	0.49 ± 0.39, /, (0.02, 1.92)	1.30 ± 1.00, /, (0.42, 4.38)	1.12 ± 1.01, /, (0.17, 4.19)	3.68 ± 3.66, /, (0.23, 16.21)	0.49 ± 0.39, /, (0.03, 1.39)	/
		0.75 ± 0.33, /, (0.23, 1.67)	0.63 ± 0.35, /, (0.21, 1.66)	1.24 ± 0.85, /, (0.21, 3.83)	1.15 ± 0.88, /, (0.20, 3.81)	3.55 ± 2.97, /, (0.12, 12.65)	0.31 ± 0.24, /, (0.01, 0.94)	/
24	Yuan Chen (2020) [43]	0.59 ± 0.28, /, (0.10, 1.30)	/	0.99 ± 0.41, /, (0.20, 1.80)	/	1.91 ± 1.02, /, (0.20, 4.20)	0.38 ± 0.26, /, (0.00, 1.10)	1
		1.04 ± 0.64, /, (0.10, 3.10)	/	1.46 ± 0.64, /, (0.50, 3.30)	/	2.77 ± 1.72, /, (0.40, 6.30)	0.84 ± 0.68, /, (0.10, 3.10)	0
25	Dong Wu (2020) [44]	1.22 ± 0.70, /, /	/	1.33 ± 0.73, /, /	/	4.34 ± 2.22, /, /	/	/
26	Kang-jie Cheng (2020) [45]	0.79 ± 0.17, /, /	0.61 ± 0.19, /, /	1.26 ± 0.27, /, /	0.91 ± 0.55, /, /	3.77 ± 1.57, /, /	(coronal)0.38 ± 0.17, /, / (apical)0.37 ± 0.20, /, /	/
27	Pantip Henprasert (2020) [46]	0.27 ± 0.12, /, /	/	0.81 ± 0.28, /, /	/	(mesio-distal) 0.77 ± 0.72, /, / (bucco-lingual) 1.77 ± 0.76, /, /	(buccal)0.21 ± 0.24, /, / (lingual)0.23 ± 0.12, /, / (distal)0.40 ± 0.32, /, / (mesial)0.33 ± 0.37, /, /	/
		0.32 ± 0.15, /, /	/	0.84 ± 0.47, /, /	/	(mesio-distal) 0.78 ± 0.80, /, / (bucco-lingual) 1.60 ± 1.22, /, /	(buccal)0.24 ± 0.23, /, / (lingual)0.25 ± 0.17, /, / (distal)0.33 ± 0.23, /, / (mesial)0.37 ± 0.28, /, /	/
28	Kristian Kniha (2020) [47]	1.47 ± 0.86, /, (0.0, 3.40)	/	1.77 ± 0.85, /, (0.50, 3.20)	/	2.81 ± 2.69, /, (0.00, 8.40)	0.10 ± 0.46, /, (− 0.70, 0.90)	/
		1.47 ± 0.86, /, (0.0, 3.40)	/	1.77 ± 0.85, /, (0.50, 3.20)	/	2.81 ± 2.69, /, (0.00, 8.40)	− 0.07 ± 0.54, /, (− 0.90, 1.00)	/
		1.31 ± 0.61, /, (0.10, 2.60)	/	1.91 ± 0.79, /, (0.50, 3.10)	/	6.22 ± 4.26, /, (0.00, 15.30)	0.22 ± 0.58, /, (− 1.00, 1.10)	/
		1.31 ± 0.61, /, (0.10, 2.60)	/	1.91 ± 0.79, /, (0.50, 3.10)	/	6.22 ± 4.26, /, (0.00, 15.30)	− 0.31 ± 0.66, /, (− 1.40, 0.90)	/
29	R. Vinci (2020) [48]	/	0.67 ± 0.37, /, (0.30, 1.77)	/	0.89 ± 0.30, /, (0.10, 1.57)	/	/	3
		/	0.12 ± 0.28, /, (0.08, 1.18)	/	0.31 ± 0.43, /, (0.30, 1.77)	/	/	3
		/	0.41 ± 0.31, /, (0.08, 1.30)	/	0.88 ± 0.44, /, (0.17, 2.66)	/	/	3
		/	0.31 ± 0.38, /, (0.27, 1.77)	/	0.79 ± 0.40, /, (0.10, 3.54)	/	/	3
30	Nopparat Suksod (2020) [49]	0.98 ± 0.48, /, /	/	1.57 ± 0.46, /, /	/	4.23 ± 1.84, /, /	/	/
31	Márton Kivovics (2020) [50]	1.987 ± 0.7049, /, /	/	1.954 ± 0.6853, /, /	/	6.544 ± 5.393, /, /	/	3
		1.879 ± 0.7893, /, /	/	2.124 ± 0.8373, /, /	/	7.177 ± 4.214, /, /	/	3
32	Palita Smitkarn (2019) [51]	1.0 ± 0.6, 0.9, (0.20, 2.67)	/	1.3 ± 0.6, 1.2, (0.24, 2.57)	/	3.1 ± 2.3, 2.8, (0.00, 8.60)	/	/
33	Karim El Kholy (2019) [52]	0.284 ± 0.133, /, (0.051, 0.583)	/	0.675 ± 0.429, /, (0.140, 1.980)	/	4.363 ± 1.682, /, (1.180, 8.800)	/	/
		1.015 ± 0.124, /, (0.840, 1.230)	/	1.657 ± 0.209, /, (1.310, 1.940)	/	7.713 ± 1.236, /, (5.500, 10.500)	/	/
		0.562 ± 0.086, /, (0.410, 0.710)	/	1.195 ± 0.397, /, (0.850, 1.920)	/	5.688 ± 1.521, /, (3.200, 8.300)	/	/
		0.289 ± 0.159, /, (0.060, 0.591)	/	0.616 ± 0.255, /, (0.220, 1.000)	/	4.731 ± 1.601, /, (2.600, 8.500)	/	/
34	Henrik Skjerven (2019) [53]	1.05 ± 0.59, /, (0.36, 2.74)	/	1.63 ± 1.05, /, (0.56, 5.16)	/	3.85 ± 1.83, /, (1.25, 8.60)	0.48 ± 0.5, /, (0.52, 1.34)	/
35	Rai-Jei Chang (2018) [54]	/, 0.40, (0.00, 1.00)	/	/, 0.65, (0.10, 1.90)	/	/, 2.16, (0.17, 6.91)	/	1
		/, 0.95, (0.30, 1.30)	/	/, 1.35, (0.10, 3.60)	/	/, 3.92, (0.44, 11.66)	/	1
36	Zhaozhao Chen (2018) [55]	0.85 ± 0.38, /, (0.42, 1.51)	/	0.93 ± 0.34, /, (0.64, 1.72)	/	3.11 ± 1.55, /, (0.66, 4.95)	(Considering direction) -0.32 ± 0.48, /, (− 1.00, 0.64) (Absolute value) 0.50 ± 0.26, /, (0.18, 1.00)	/
37	Jan Brandt (2018) [56]	/	0.725 ± 0.142, /, (0.518, 1.112)	/	0.990 ± 0.244, /, (0.633, 1.526)	2.011 ± 0.855, /, (0.366, 4.036)	0.541 ± 0.129, /, (0.242, 0.848)	/
38	Philipp Kauffmann (2018) [57]	/, 0.47, (0.05, 1.31)	/	/, 0.86, (0.21, 1.68)	/	/, 3.41, (0.48, 5.79)	/, 0.44, (0.03, 1.54)	3
		/, 0.49, (0.10, 1.11)	/	/, 0.77, (0.16, 1.86)	/	/, 2.76, (0.32, 7.54)	/, 0.52, (0.06, 1.69)	0
39	Yiqin Fang (2018) [58]	0.46, /, (0.00, 1.15)	/	0.67, /, (0.14, 1.19)	/	1.40, /, (0.30, 2.57)	0.15, /, (0.10, 0.82)	/
40	Boyoung Ma (2018) [59]	0.82 ± 0.44, /, (0.13, 1.85)	/	1.19 ± 0.46, /, (0.37, 2.51)	/	2.43 ± 1.13, /, (1.15, 5.70)	-0.03 ± 0.65, /, (-2.12, 1.58)	/
		1.37 ± 0.80, /, (0.18, 3.76)	/	1.77 ± 0.86, /, (0.45, 3.76)	/	4.74 ± 2.06, /, (0.00, 8.86)	/	/
41	Björn Gjelvold (2018) [60]	0.27 ± 0.08, /, /	/	0.34 ± 0.14, /, /	/	0.99 ± 0.57, /, /	0.16 ± 0.11, /, /	/
		0.39 ± 0.01, /, /	/	0.49 ± 0.17, /, /	/	1.25 ± 0.49, /, /	0.34 ± 0.18, /, /	/

**Table 3:**
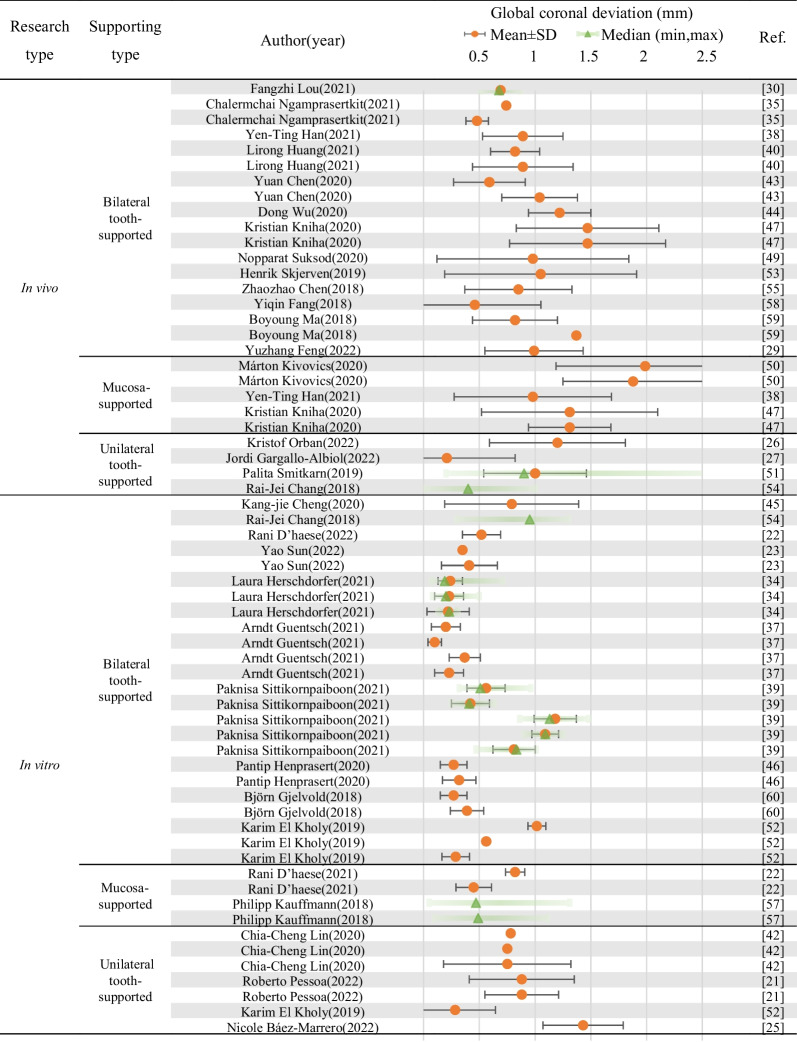
Forest plot showing the global coronal deviations of the reviewed studies concerning different guide supporting types in different research types

**Table 4:**
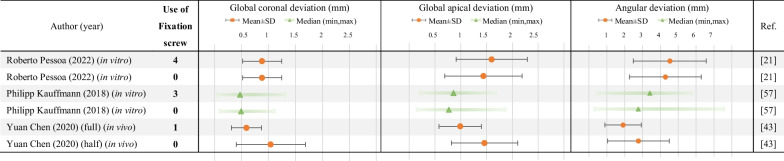
Forest plot showing the global coronal deviations, global apical deviations and angular deviations of the reviewed studies concerning fixation screws application

**Table 5:**
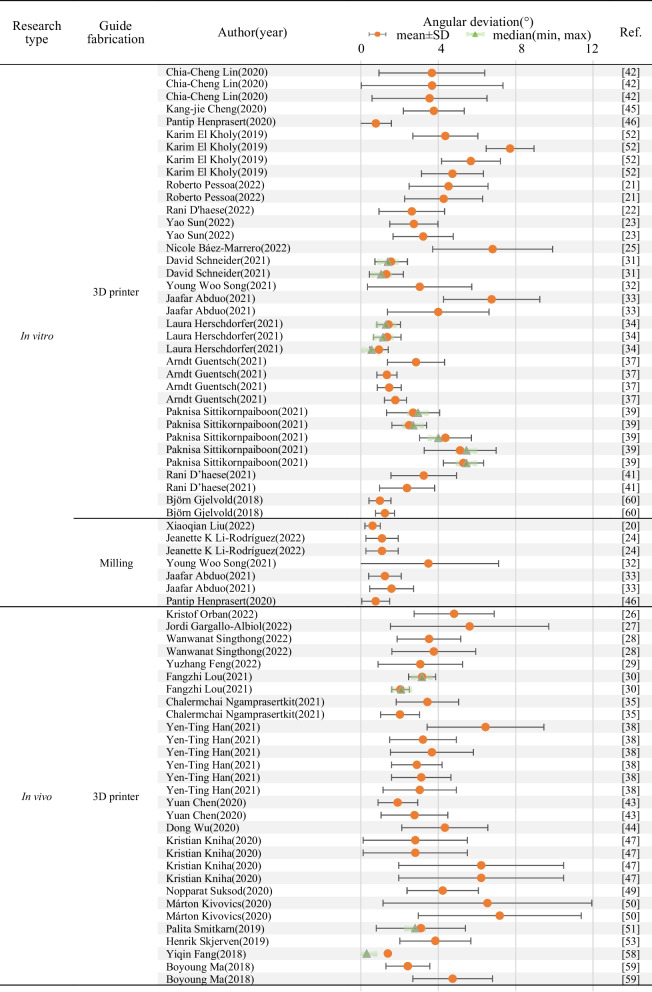
Forest plot showing the angular deviations of the reviewed studies concerning different guide fabrication strategies in different research types

As presented in Table 3 and Additional file 1: Tables S1, S2 forest plots of global coronal deviations, global apical deviations and angular deviations from the reviewed studies are summarized according to different guide supporting types. Tooth-supported guides are the most frequently applied in studies published in recent five years: among the 41 involved literatures, 27 involved bilateral tooth-supported guides, 9 involved mixed tooth-/bone- or tooth-/mucosa-supported guides (unilateral tooth-supported), and only 7 applied mucosa-supported guides. The accuracy-related numerical data (mean value, or median when mean value is not available) of bilateral tooth-supported implant guide ranges within 0.1–1.18 mm (in vitro)/0.46–1.47 mm (in vivo) in global coronal deviation, 0.18 ~ 1.37 mm (in vitro)/0.39–1.07 mm (in vivo) in horizontal coronal deviation, 0.12–1.95 mm (in vitro)/0.28–1.77 mm (in vivo) in global apical deviation, 0.31–1.68 mm (in vitro)/0.64–1.17 mm (in vivo) in horizontal apical deviation, 0.77–7.713° (in vitro)/1.4–4.74° (in vivo) in angular deviation, and 0.11–0.95 mm (in vitro)/0.03–0.84 mm (in vivo) in vertical deviation. Global coronal deviations of unilateral tooth-supported guides (including mixed tooth-/bone- or tooth-/mucosa-supported guides) are between 0.284–1.43 mm (in vitro)/0.21–1.2 mm (in vivo), horizontal coronal deviations between 0.47–0.67 mm (in vitro), global apical deviations between 0.65–2.19 mm (in vitro)/0.67–1.45 mm (in vivo), horizontal apical deviations between 0.91–1.72 mm (in vitro), angular deviations between 2.16–6.81 mm (in vitro)/3.1–5.62 mm (in vivo)°, and vertical deviations between 0.16–0.57 mm (in vitro). Global coronal deviations of mucosa-supported guides are between 0.45–0.82 mm (in vitro)/0.98–1.987 mm (in vivo), horizontal coronal deviations between 0.12–0.67 mm (in vivo), global apical deviations between 0.71–0.99 mm (in vitro)/1.18–2.124 mm (in vivo), horizontal apical deviations between 0.31–0.89 mm (in vivo), angular deviations between 2.39–3.41° (in vitro)/3.12–7.177° (in vivo), and vertical deviations between 0.44–0.52 mm (in vitro)/0.22–0.64 mm (in vivo) (Fig. 4, more data see Table 3).

Only 3 studies involved the comparison of guides with/without fixation screws, among which 2 were in vitro studies, and 1 was in vivo study (Table 4). Only 2 studies involved the comparison of sleeve length of guides.

Among the 41 involved literatures in this review, 29 involved 3D printing-fabricated guides, 3 involved milling-fabricated guides, 3 involved both, and 6 involved no information of fabricating methods. The in vitro accuracy-related numerical data (mean value, or median when mean value is not available) of milling guides ranges within 0.2–1.37 mm in horizontal coronal deviation, 0.62–1.68 mm in horizontal apical deviation, 0.61–3.49° in angular deviation; and the in vitro data of 3d printing guides ranges within 0.1–1.43 mm in global coronal deviation, 0.18–0.95 mm in horizontal coronal deviation, 0.12–2.19 mm in global apical deviation, 0.31–1.72 mm in horizontal apical deviation, 0.78–7.713° in angular deviation., and 0.11–0.64 mm in vertical deviation. The in vivo data of milling guides ranges within 0.12–0.67 mm in horizontal coronal deviation, 0.31–0.89 mm in horizontal apical deviation; and the data of 3d printing guides ranges within 0.21–1.987 mm in global coronal deviation, 0.39–1.12 mm in horizontal coronal deviation, 0.28–2.24 mm in global apical deviation, 0.64–1.57 mm in horizontal apical deviation, 1.4–7.177° in angular deviation, and 0.03–1.26 mm in vertical deviation (Fig. 5, more data see Table 2). Studies of milled guides mainly apply the horizontal deviation indicators following its coordinate system, less than 3 literatures apply the global deviation indicators, therefore the forest plots (Table 5 and Supplementary Table S3-S4) and normal distributions (Fig. 5) concerning different fabrication approaches are based on horizontal coronal deviations and horizontal apical deviations.

As visually shown in Tables 3, 4, 5 and Additional file 1: Tables S1–S4, with current technology of digital implant guides, implant surgery accuracy can be achieved with the mean distance deviation < 2 mm (most < 1 mm) and angular deviation < 8° (most < 5°).

## Discussion

### Guide supporting type

**Fig. 3:**
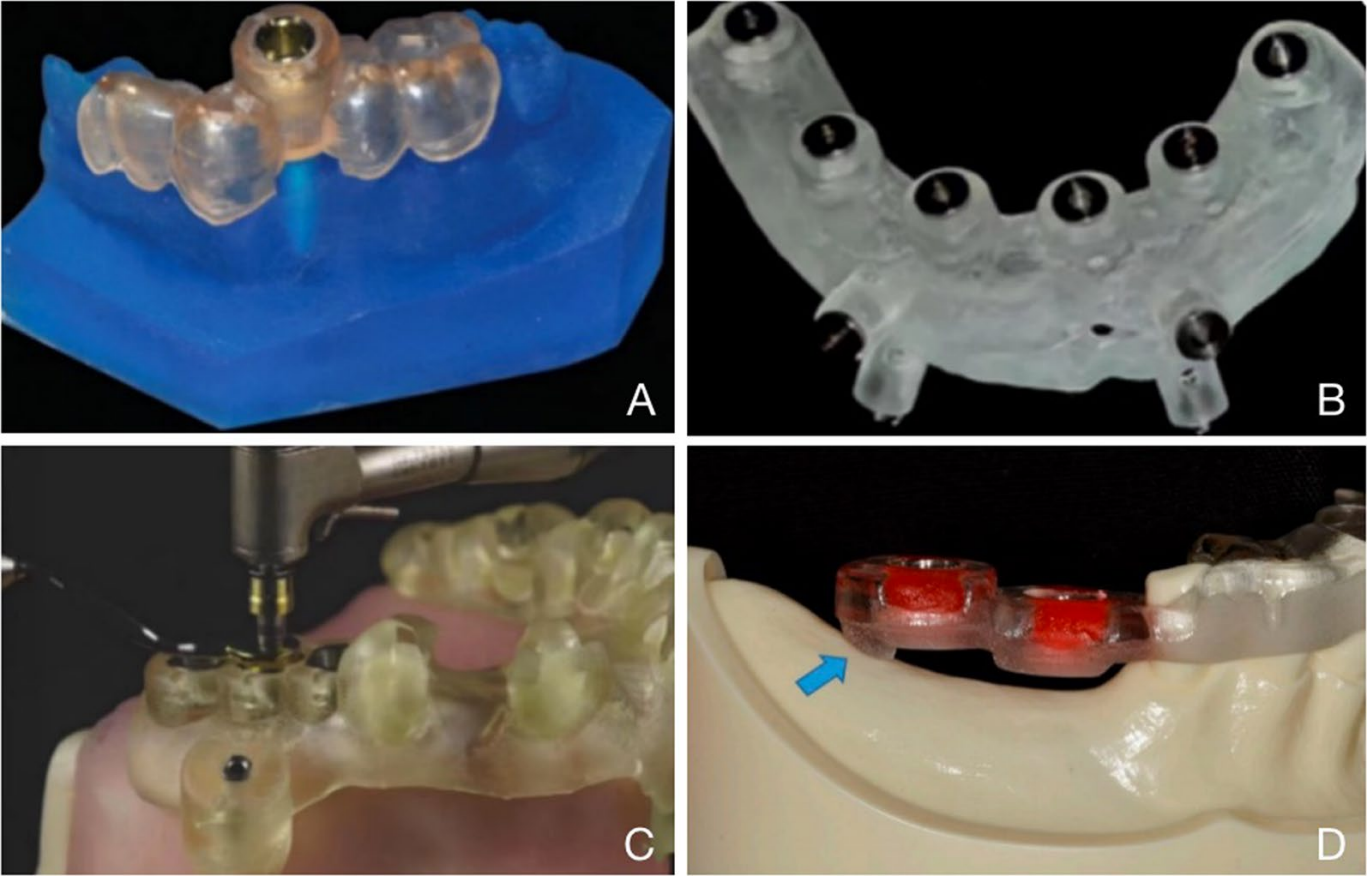
Different support types of implanting guides: **A** bilateral tooth-supported [30]; **B** mucosa-supported [61]; **C** mixed tooth-/mucosa-supported [35]; **D** mixed tooth-/bone-supported [42]

To verify the hypothesis that supporting types influence the accuracies of surgical guides, we collectively categorized and analyzed guide type and deviation data in existing literature. Implant guides are divided into categories according to its support types, including bone-supported, mucosa-supported, tooth-supported, and any combination (Fig. 3). Theoretically, the anatomical differences among teeth, bone and mucosa may lead to different accuracy of guides with different support types. Bilateral tooth-supported guides provide best retention and biomechanical stability with anchorage on hard tissue, therefore theoretically endow highest accuracy.

Although with advantages in accuracy and operability, bilateral tooth-supported guides are indicated for patients with intact teeth both mesial and distal to the edentulous area. As for distal extension edentulism, one of the most common clinical manifestation, unilateral tooth-supported guides including mixed tooth-/bone- or tooth-/mucosa-supported guides are often used to provide efficient retention.

Bone-supported guides are overlaid on the alveolar crest exposed via full‐thickness mucoperiosteal flap operation and fixed with fixation screws. Its larger surgical wound, upturned tissue flap affects its repositioning, resulting in relatively low theoretical accuracy. Simple bone-supported guides are seldom reported in recent five years [62], and among the 41 researches included in this review, only two studies applied mixed tooth-/bone-supported guides [63, 64].

Mucosa-supported guides are indicated for completely edentulous patients or patients who barely have residual teeth. Without flap operation, it is anchored to the bone through the mucosa with fixation screws. To be noted, a recent research reported that calculation of implant angular deviation of mucosa-supported guides by tissue or implant alignment resulted in different values [41], emphasizing the lack of standard for accuracy measurement, and indicating that comparability of the accuracy indicator values in different literatures should be reviewed dialectically.

Since different guide support types are used in various situations, the existing literature rarely directly compares the accuracy among different guide types. A systematic review in 2012 compares the accuracy of different guide types, and reveals a significant higher accuracy of tooth- and mucosa-supported guides than bone-supported ones, with no significant difference between tooth- and mucosa-supported guides [63]. The review mentions only two researches that directly compare different support types [65, 66]. A systematic review in 2018 showed that the implantation placement in cases using tooth-supported guides is more accurate than bone- and mucosa-supported [16].

**Fig. 4:**
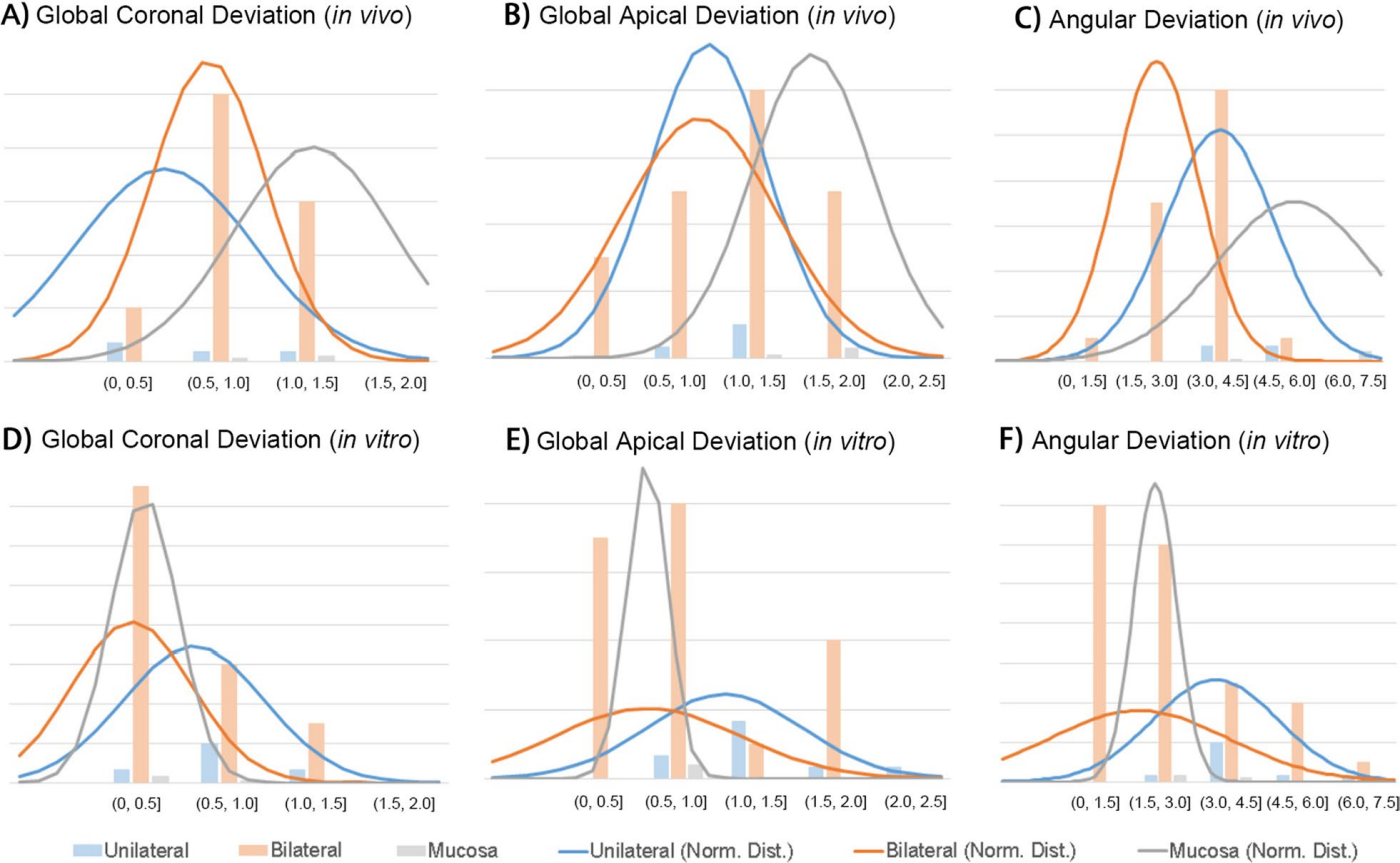
Normal distributions of global coronal deviations, global apical deviations, and angular deviations of the reviewed studies concerning different guide supporting types in different research types

In this review, as accuracy-related parameters differ in different studies, three parameters with relatively high frequency of application (global coronal deviations, global apical deviations and angular deviations) are selected for normal distribution analyses (Fig. 4). It is interesting to notice that the normal distribution of deviations varied with research types, especially for mucosa-supported guides, which exhibited the highest deviation peaks in all three parameters in in vivo researches (Fig. 4A–C), and the lowest in deviation peaks and narrowest distribution in vitro studies (Fig. 4D–F). This opposite trend was probably attributed to the different elasticity between in vitro experimental model and in vivo mucosa. In vitro experimental mucosa-imitating models exhibited simpler mechanical properties than living tissues, resulting in higher accuracies.

In addition, the use of anesthetics may affect the accuracy of the procedure due to elasticity of mucosa and its deformation upon penetration [67]. Compared to bilateral tooth-supported guides, unilateral tooth guides exhibited higher deviations in most categories (Fig. 4C–F), except in in vivo global coronal deviation where unilateral tooth-supported guides exhibited slightly lower distribution than bilateral guides (Fig. 4A), and in in vivo global apical deviation where bilateral and unilateral tooth-supported guides showed a similar data range (Fig. 4B).

In summary, bilateral tooth-supported guides exhibited the highest in vitro accuracy and similar in vivo accuracy to unilateral tooth-supported guides; mucosa-supported guides exhibit the lowest in vivo accuracy, while its in vitro data showed low credibility due to the mechanical complexity of living mucosa tissue.

### Design of fixation screws

For bone- and mucosa-supported guides, fixation screws can be further introduced to fix the surgical guide and avoid displacement. The accuracy of implantation is reported to be improved by the application of fixation screws and influenced by its distribution [63, 68]. Since fixation screws are regular designs for implant guides, guides without fixation screws are only involved in three studies [21, 43, 57] as included in this review, all compared to groups with fixation screws in the same research. The difference between the two in vitro studies [21, 57] was not significant, and though the in vivo study [43] reported significantly higher accuracy in experimental group using fixation screws, whether the accuracy was influenced by the guide type (full-/half-) or the usage of fixation screws still requires further study due to the small sample size (Table 4).

For mucosa-supported guides used in edentulous patients, the use of fixation screws provide larger surface support and reduce the intraoperative displacement [69], efficiently reduce the angular deviation [68], depth deviation and horizontal deviation [57]. Therefore, in cases demanding a high depth precision and avoiding injury to the mandibular nerve, application of fixation screws contribute to better implant results [57].

For mixed tooth-/mucosa-supported guide in free-end dental implantation, application of fixation screws also results in a significant improvement in the accuracy regarding horizontal apical and depth deviation (direction considered) [21]. Apart from mucosa-supported and mixed tooth-/mucosa-supported guides, fixation screws can also be introduced into tooth-supported guides to achieve improved stability in both maxillary and mandibular anterior implantation [40, 43].

Concerning the number of fixation screws, most design applied three-point fixation [48, 50, 57], though the number can be adjusted [21, 25, 40, 43, 54]. A systematic review in 2012 indicates that when fixation screws are used, the mean deviation for all indicators are reduced, and the deviations decrease as the number of fixation screws increases, but the indicators exhibit no significant difference [63]. Design of two equally distributed fixation screws can also efficiently stabilize the surgical guide, located approximately at the lateral incisor [70]. Ideally, for fully edentulous maxillae, four-point fixation (two in anterior area and distal to the implant site) that covers the entire maxillary arch can efficiently avoid bending of the guide in the distal areas of the surgical field [71].

### Design of sleeve

The guidance of drill hole, implant direction, depth, and angle are realized via design of sleeves, which can also reduce surgical time [72–74]. Sleeves can be classified as open or closed. Open sleeves with C-shaped buccally opening are applied in posterior areas where mouth opening and interarch space are limited or insufficient [74]. To ensure implant accuracy, the drill should be in the center and parallel to the inner wall of sleeves during hole preparation [75]. As summarized in Table 2, among the 41 involved literatures in this review, only 2 involved the comparison of sleeve design of guides, indicating that smaller distance from sleeve to bone leads to more accurate results [37], and that sleeve design might affect the accuracy [39].

Implant accuracy is affected by the design of height, drilling distance, and sleeve–bone distance, but the sleeve–implant distance and the sleeve axis angle do not affect the accuracy of digital implant guides [20]. By using shorter sleeve heights or shorter implants, decreasing the drilling distance below the guided sleeve can significantly increase the implant accuracy and reduce lateral movement of the drill [76]. However, sleeve heights ≤ 5 mm lead to implant placement deviation and decrease of the accuracy [77]. The increased drilling distance beyond the guiding sleeve results in a significant global and angular deviation at both the implant crest and apex [76]. Decreased sleeve–bone distance results in higher accuracy of the implant surgical guide. With the sleeve–bone distance of 2 or 4 mm, the implant accuracy of closed and open sleeve is similar; whereas with the sleeve–bone distance of 6 mm, lower accuracy is shown in both open and closed sleeves, and open sleeves exhibited a more significant trend [78].

In addition, material of the sleeve also affects implant accuracy. Metal sleeves are common in early surgery guides, and with the development of material science and technology, it is reported that plastic sleeves endow lower angle deviation, depth deviation, placement deviation than metal ones, as well as ensure a faster and easier guided surgery work-flow [79].

### Manufacturing accuracy

Currently, digital implant guides can be manufactured using additive manufacturing (3D printing) or subtractive manufacturing (CAD/CAM milling). Typical 3D printing includes stereo lithography appearance (SLA), PolyJet, MultiJet, fused filament fabrication (FFF), digital light processing (DLP), etc. The difference in manufacturing accuracy between these two strategies remains inconclusive. It has been reported that the processing accuracy of milling (0.02–0.25 mm) is higher than printing (0.03–0.44 mm) in the aspect of inner surface, vertical fit discrepancy, guide seating distortion, and error range of anterior and posterior implants [80]. However, some literatures indicated no significant difference in manufacturing accuracy between milling and 3D printing [81]. Difference of manufacturing accuracy among different 3D printing manufacturing technologies has also been reported. For example, DLP printer exhibited a lower manufacturing error compared to FFF printer [80].

Despite of the differences in manufacturing accuracy, clinical researches indicated no significant differences in all surgical accuracy indicators between additive and subtractive manufactured guides [46]. Implant guides manufactured by different additive technologies (SLA, PolyJet and MultiJet) exhibited similar surgical accuracy regarding the angle, coronal and apical deviation [34]. DLP printer showed higher accuracy than SLA printer in coronal and vertical deviation, and no significant difference in apical, horizontal and angle deviation [60].

**Fig. 5:**
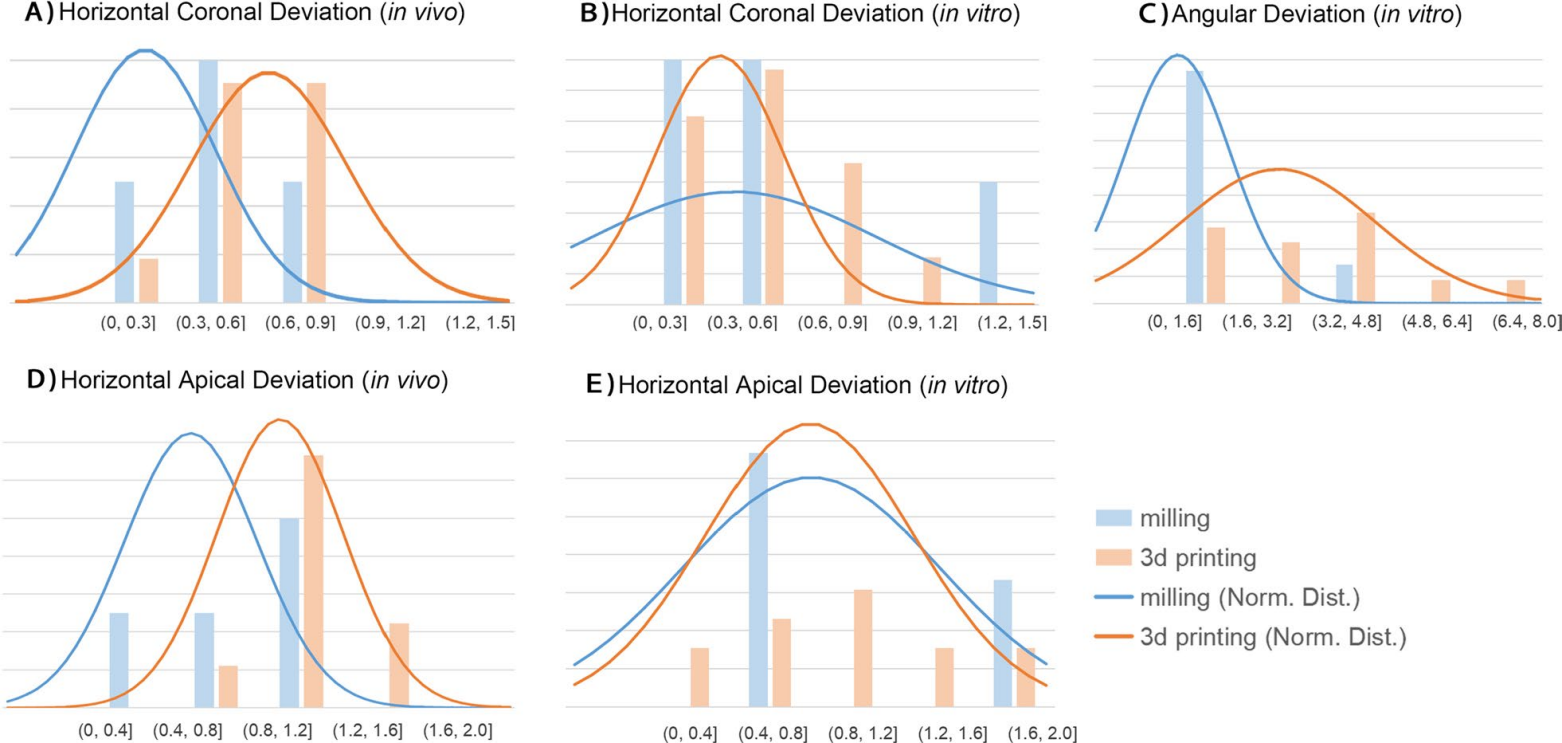
Normal distributions of horizontal coronal deviations, horizontal apical deviations, and angular deviations of the reviewed studies concerning different fabrication approaches in different research types

As shown in Table 5 and Additional file 1: Tables S3–S4, in terms of surgical guide fabrication, 3d printing is of wider application than milling. As indicated in Fig. 5, among the included literatures in this review, 3D printed guides exhibited higher peaks of horizontal coronal and horizontal apical deviations in in vivo researches than milled guides (Fig. 5A, D, in vivo angular deviation data were not sufficient to perform normal distribution analysis herein). While in in vitro researches, different trends were observed in the three deviation parameters (Fig. 5B, C, E).

In summary, though milled guides exhibited higher in vivo accuracy than 3d printed guides, the small sample size of milled guides reviewed in this article resulted in relatively low reliability of this conclusion, and further data support might be needed.

## Conclusion and prospect

This review has verified the hypothesis that guide supporting types, manufacturing methods and design of implant guides (including fixation screws and sleeves) could influence the accuracies of static digital surgical guides by collecting and categorizing the numerical data of surgical accuracy indicator reported in literature of the last five years. Bilateral tooth-supported guides exhibited the highest in vitro accuracy and similar in vivo accuracy to unilateral tooth-supported guides; mucosa-supported guides exhibit the lowest in vivo accuracy, while its in vitro data showed low credibility due to the mechanical complexity of living mucosa tissue. Milled guides exhibited higher in vivo accuracy than 3d printed guides, though further data support might be needed. Apart from operator's skill and standardization that may affect the accuracy of implantation, this review has revealed that with current medical technology and the aid of digital implant guides, implant surgery accuracy can be achieved with the distance deviation < 2 mm (most < 1 mm) and angular deviation < 8° (most < 5°). The bottleneck of surgical accuracy improvement resulted from the difficulty of guide fixation in edentulous patients. In addition, the lack of a universal evaluation standard for implantation surgical accuracy remained a major problem in this research field.

As the design of supporting types, fixation screws and sleeves of implant guides can affect the accuracy of implant surgeries, existing studies focus on improving the accuracy via selecting appropriate supporting types, optimizing and customizing the guide design (including fixation screws and sleeves) according to individual demands. Future developing trend of this field may continuously focus on standardization of the evaluation of surgical accuracy and improving minimally invasive surgical methods (such as gradually phasing out bone-support guides that involve flap surgeries). The improvement of implant accuracy for edentulous patients has been a field of intense research in recent years and may remain a research focus in the near future.

The influence of implant guide design on surgical accuracy revealed in this review may shed light on future improvement of digital implant guides. However, this review only analyzed and discussed four influencing factors that affected the implantation accuracy, other factors including the guiding protocol (full/half guide), implant position (maxillary/mandibular, anterior/posterior, etc.), implant size, bone quality, etc., remained undiscussed and required further analysis in future reviews.

### Supplementary Information

**Additional file 1: **Methods.** Figure S1.** Risk of bias of included observational studies. **Table S1. **Forest plot showing the global apical deviations of the reviewed studies concerning different guide supporting types in different research types. **Table S2**. Forest plot showing the angular deviations of the reviewed studies concerning different guide supporting types in different research types. **Table S3. **Forest plot showing the horizontal coronal deviations of the reviewed studies concerning different guide fabrication in different research types. **Table S4. **Forest plot showing the horizontal apical deviations of the reviewed studies concerning different guide fabrication in different research types.

## Data Availability

The data that support the findings of this study are available from the corresponding author upon reasonable request.

## Abbreviations

AR: Augmented reality
CBCT: Cone beam CT
ISO: International Organization for Standardization
IOSs: Intraoral scanners
PICO: Participants, Intervention, Comparison or control and Outcome measures
3D: Three-dimensional
ID: Identity document
NOS: Newcastle–Ottawa Scale
WOS: Web of Science
SD: Standard deviation
n: Number of records
CAD: Computer Aided Design
CAM: Computer Aided Manufacturing
SLA: Stereo lithography appearance
FFF: Fused filament fabrication
DLP: Digital light processing
Ref.: Reference
No.: Numero sign/numero symbol
SUMHS: Shanghai University of Medicine & Health Sciences
