# Supplementary material for: A systematic review of the accuracy of digital surgical guides for dental implantation
Source: Int J Implant Dent. 2023 Oct 25;9:38. doi: 10.1186/s40729-023-00507-w (PMC10597938; doi:10.1186/s40729-023-00507-w)
Supplement: Supplementary file 1 — Additional file 1: Methods. Figure S1. Risk of bias of included observational studies. Table S1. Forest plot showing the global apical deviations of the reviewed studies concerning different guide supporting types in different research types. Table S2. Forest plot showing the angular deviations of the reviewed studies concerning different guide supporting types in different research types. Table S3. Forest plot showing the horizontal coronal deviations of the reviewed studies concerning different guide fabrication in different research types. Table S4. Forest plot showing the horizontal apical deviations of the reviewed studies concerning different guide fabrication in different research types. [file 40729_2023_507_MOESM1_ESM.docx]

**Methods**

**The Newcastle–Ottawa Scale (NOS) adapted by Chambrone et al. (2010, 2015)**

1. Selection of study groups

a) Sample size calculation

b) Representativeness and selection of the

patients submitted to guided surgery

c) Clear description of the all steps for guided

surgery

d) Training/calibration of the surgeons and

assessors of outcomes

e) Data collection: preoperative and postoperative CT scans

f) Description of clear inclusion/exclusion criteria

2. Comparability

a) Comparability of patients on the basis of the study design or analysis: patients who need a mucosa-, tooth-, or bone-supported guide

b) Management of potential confounders

3. Outcome

a) Evaluation of results

b) Assessment of accuracy outcomes

c) Adequacy of follow-up of the patients

4. Statistical analysis; all of the studies have adequacy statistical analysis

a) Appropriateness/validity of statistical analysis

b) Unit of analysis reported in the statistical model

**Figure S1.** Risk of bias of included observational studies

**Table S1.** Forest plot showing the global apical deviations of the reviewed studies concerning different guide supporting types in different research types.

| Research type | Supporting type | Author (year) | Global apical deviation (mm)  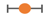Mean±SD 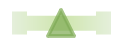Median (min,max)  0.5 1 1.5 2 2.5 3 | Ref. |
| --- | --- | --- | --- | --- |
| *In vivo* | bilateral tooth-supported | Fangzhi Lou(2021) |  | [1] |
|  |  | Fangzhi Lou(2021) |  | [1] |
|  |  | Chalermchai Ngamprasertkit(2021) |  | [2] |
|  |  | Chalermchai Ngamprasertkit(2021) |  | [2] |
|  |  | Yen-Ting Han(2021) |  | [3] |
|  |  | Lirong Huang(2021) |  | [4] |
|  |  | Lirong Huang(2021) |  | [4] |
|  |  | Yuan Chen(2020) |  | [5] |
|  |  | Yuan Chen(2020) |  | [5] |
|  |  | Dong Wu(2020) |  | [6] |
|  |  | Kristian Kniha(2020) |  | [7] |
|  |  | Kristian Kniha(2020) |  | [7] |
|  |  | Nopparat Suksod(2020) |  | [8] |
|  |  | Henrik Skjerven(2019) |  | [9] |
|  |  | Zhaozhao Chen(2018) |  | [10] |
|  |  | Yiqin Fang(2018) |  | [11] |
|  |  | Boyoung Ma(2018) |  | [12] |
|  |  | Boyoung Ma(2018) |  | [12] |
|  |  | Björn Gjelvold(2018) |  | [13] |
|  |  | Björn Gjelvold(2018) |  | [13] |
|  |  | Yuzhang Feng(2022) |  | [14] |
|  | mucosa-supported | Yen-Ting Han(2021) |  | [3] |
|  |  | Kristian Kniha(2020) |  | [7] |
|  |  | Kristian Kniha(2020) |  | [7] |
|  |  | Márton Kivovics(2020) |  | [15] |
|  |  | Márton Kivovics(2020) |  | [15] |
|  | unilateral tooth-supported | Palita Smitkarn(2019) |  | [16] |
|  |  | Kristof Orban(2022) |  | [17] |
|  |  | Jordi Gargallo-Albiol(2022) |  | [18] |
|  |  | Rai-Jei Chang(2018) |  | [19] |
| *In vitro* | bilateral tooth-supported | Rani D'haese(2022) |  | [20] |
|  |  | Yao Sun(2022) |  | [21] |
|  |  | Yao Sun(2022) |  | [21] |
|  |  | Laura Herschdorfer(2021) |  | [22] |
|  |  | Laura Herschdorfer(2021) |  | [22] |
|  |  | Laura Herschdorfer(2021) |  | [22] |
|  |  | Arndt Guentsch(2021) |  | [23] |
|  |  | Arndt Guentsch(2021) |  | [23] |
|  |  | Arndt Guentsch(2021) |  | [23] |
|  |  | Arndt Guentsch(2021) |  | [23] |
|  |  | Paknisa Sittikornpaiboon(2021) |  | [24] |
|  |  | Paknisa Sittikornpaiboon(2021) |  | [24] |
|  |  | Paknisa Sittikornpaiboon(2021) |  | [24] |
|  |  | Paknisa Sittikornpaiboon(2021) |  | [24] |
|  |  | Paknisa Sittikornpaiboon(2021) |  | [24] |
|  |  | Pantip Henprasert(2020) |  | [25] |
|  |  | Pantip Henprasert(2020) |  | [25] |
|  |  | Karim El Kholy(2019) |  | [26] |
|  |  | Karim El Kholy(2019) |  | [26] |
|  |  | Karim El Kholy(2019) |  | [26] |
|  | mucosa-supported | Rani D’haese(2021) |  | [27] |
|  |  | Rani D’haese(2021) |  | [27] |
|  |  | Philipp Kauffmann(2018) |  | [28] |
|  |  | Philipp Kauffmann(2018) |  | [28] |
|  | unilateral tooth-supported | Chia-Cheng Lin(2020) |  | [29] |
|  |  | Chia-Cheng Lin(2020) |  | [29] |
|  |  | Chia-Cheng Lin(2020) |  | [29] |
|  |  | Roberto Pessoa(2022) |  | [30] |
|  |  | Roberto Pessoa(2022) |  | [30] |
|  |  | Nicole Báez-Marrero(2022) |  | [31] |
|  |  | Kang-jie Cheng(2020) |  | [32] |
|  |  | Rai-Jei Chang(2018) |  | [19] |
|  |  | Karim El Kholy(2019) |  | [26] |

**Table S2**. Forest plot showing the angular deviations of the reviewed studies concerning different guide supporting types in different research types.

| Research type | Supporting type | Author(year) | Angular deviation (mm)  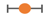Mean±SD 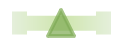Median (min,max)  0 2 4 6 8 10 | | Ref. |
| --- | --- | --- | --- | --- | --- |
| *In vivo* | bilateral tooth-supported | Yuzhang Feng(2022) | |  | [14] |
|  |  | Wanwanat Singthong(2022) | |  | [33] |
|  |  | Wanwanat Singthong(2022) | |  | [33] |
|  |  | Fangzhi Lou(2021) | |  | [1] |
|  |  | Fangzhi Lou(2021) | |  | [1] |
|  |  | Chalermchai Ngamprasertkit(2021) | |  | [2] |
|  |  | Chalermchai Ngamprasertkit(2021) | |  | [2] |
|  |  | Yen-Ting Han(2021) | |  | [3] |
|  |  | Lirong Huang(2021) | |  | [4] |
|  |  | Lirong Huang(2021) | |  | [4] |
|  |  | Yuan Chen(2020) | |  | [5] |
|  |  | Yuan Chen(2020) | |  | [5] |
|  |  | Dong Wu(2020) | |  | [6] |
|  |  | Kristian Kniha(2020) | |  | [7] |
|  |  | Kristian Kniha(2020) | |  | [7] |
|  |  | Nopparat Suksod(2020) | |  | [8] |
|  |  | Henrik Skjerven(2019) | |  | [9] |
|  |  | Zhaozhao Chen(2018) | |  | [10] |
|  |  | Yiqin Fang(2018) | |  | [11] |
|  |  | Boyoung Ma(2018) | |  | [12] |
|  |  | Boyoung Ma(2018) | |  | [12] |
|  | mucosa-supported | Yen-Ting Han(2021) | |  | [3] |
|  |  | Kristian Kniha(2020) | |  | [7] |
|  |  | Kristian Kniha(2020) | |  | [7] |
|  |  | Márton Kivovics(2020) | |  | [15] |
|  |  | Márton Kivovics(2020) | |  | [15] |
|  | unilateral tooth-supported | Palita Smitkarn(2019) | |  | [16] |
|  |  | Kristof Orban(2022) | |  | [17] |
|  |  | Jordi Gargallo-Albiol(2022) | |  | [18] |
|  |  | Rai-Jei Chang(2018) | |  | [19] |
| *In vitro* | bilateral tooth-supported | Pantip Henprasert(2020) | |  | [25] |
|  |  | Pantip Henprasert(2020) | |  | [25] |
|  |  | Pantip Henprasert(2020) | |  | [25] |
|  |  | Pantip Henprasert(2020) | |  | [25] |
|  |  | Young Woo Song(2021) | |  | [34] |
|  |  | Young Woo Song(2021) | |  | [34] |
|  |  | Rani D’haese(2022) | |  | [20] |
|  |  | Yao Sun(2022) | |  | [21] |
|  |  | Yao Sun(2022) | |  | [21] |
|  |  | Jeanette K Li-Rodríguez(2022) | |  | [35] |
|  |  | Jeanette K Li-Rodríguez(2022) | |  | [35] |
|  |  | David Schneider(2021) | |  | [36] |
|  |  | David Schneider(2021) | |  | [36] |
|  |  | David Schneider(2021) | |  | [36] |
|  |  | David Schneider(2021) | |  | [36] |
|  |  | Jaafar Abduo(2021) | |  | [37] |
|  |  | Jaafar Abduo(2021) | |  | [37] |
|  |  | Jaafar Abduo(2021) | |  | [37] |
|  |  | Jaafar Abduo(2021) | |  | [37] |
|  |  | Laura Herschdorfer(2021) | |  | [22] |
|  |  | Laura Herschdorfer(2021) | |  | [22] |
|  |  | Laura Herschdorfer(2021) | |  | [22] |
|  |  | Arndt Guentsch(2021) | |  | [23] |
|  |  | Arndt Guentsch(2021) | |  | [23] |
|  |  | Arndt Guentsch(2021) | |  | [23] |
|  |  | Arndt Guentsch(2021) | |  | [23] |
|  |  | Paknisa Sittikornpaiboon(2021) | |  | [24] |
|  |  | Paknisa Sittikornpaiboon(2021) | |  | [24] |
|  |  | Paknisa Sittikornpaiboon(2021) | |  | [24] |
|  |  | Paknisa Sittikornpaiboon(2021) | |  | [24] |
|  |  | Paknisa Sittikornpaiboon(2021) | |  | [24] |
|  |  | Jan Brandt(2018) | |  | [38] |
|  |  | Björn Gjelvold(2018) | |  | [13] |
|  |  | Björn Gjelvold(2018) | |  | [13] |
|  |  | Karim El Kholy(2019) | |  | [26] |
|  |  | Karim El Kholy(2019) | |  | [26] |
|  |  | Karim El Kholy(2019) | |  | [26] |
|  | mucosa-supported | Rani D’haese(2021) | |  | [27] |
|  |  | Rani D’haese(2021) | |  | [27] |
|  |  | Johannes Spille(2021) | |  | [39] |
|  |  | Philipp Kauffmann(2018) | |  | [28] |
|  |  | Philipp Kauffmann(2018) | |  | [28] |
|  | unilateral tooth-supported | Chia-Cheng Lin(2020) | |  | [29] |
|  |  | Chia-Cheng Lin(2020) | |  | [29] |
|  |  | Chia-Cheng Lin(2020) | |  | [29] |
|  |  | Roberto Pessoa(2022) | |  | [30] |
|  |  | Roberto Pessoa(2022) | |  | [30] |
|  |  | Nicole Báez-Marrero(2022) | |  | [31] |
|  |  | Kang-jie Cheng(2020) | |  | [32] |
|  |  | Rai-Jei Chang(2018) | |  | [19] |
|  |  | Karim El Kholy(2019) | |  | [26] |

**Table S3.** Forest plot showing the horizontal coronal deviations of the reviewed studies concerning different guide fabrication in different research types.

| Research type | Guide fabrication | Author(year) | Horizontal coronal deviation(mm)  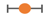Mean±SD 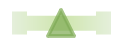Median(min,max)  0.5 1 1.5 2 | | Ref. |
| --- | --- | --- | --- | --- | --- |
| *In vitro* | 3D printer | Chia-Cheng Lin(2020) | |  | [29] |
|  |  | Chia-Cheng Lin(2020) | |  | [29] |
|  |  | Chia-Cheng Lin(2020) | |  | [29] |
|  |  | Kang-jie Cheng(2020) | |  | [32] |
|  |  | Roberto Pessoa(2022) | |  | [30] |
|  |  | Roberto Pessoa(2022) | |  | [30] |
|  |  | Yao Sun(2022) | |  | [21] |
|  |  | Yao Sun(2022) | |  | [21] |
|  |  | David Schneider(2021) | |  | [36] |
|  |  | David Schneider(2021) | |  | [36] |
|  |  | Young Woo Song(2021) | |  | [34] |
|  |  | Jaafar Abduo(2021) | |  | [37] |
|  |  | Jaafar Abduo(2021) | |  | [37] |
|  | milling | Jeanette K Li-Rodríguez(2022) | |  | [35] |
|  |  | Jeanette K Li-Rodríguez(2022) | |  | [35] |
|  |  | Young Woo Song(2021) | |  | [34] |
|  |  | Jaafar Abduo(2021) | |  | [37] |
|  |  | Jaafar Abduo(2021) | |  | [37] |
| *In vivo* | 3D printer | Kristof Orban(2022) | |  | [17] |
|  |  | Wanwanat Singthong(2022) | |  | [33] |
|  |  | Wanwanat Singthong(2022) | |  | [33] |
|  |  | Chalermchai Ngamprasertkit(2021) | |  | [2] |
|  |  | Chalermchai Ngamprasertkit(2021) | |  | [2] |
|  |  | Yen-Ting Han(2021) | |  | [3] |
|  |  | Yen-Ting Han(2021) | |  | [3] |
|  |  | Yen-Ting Han(2021) | |  | [3] |
|  |  | Yen-Ting Han(2021) | |  | [3] |
|  |  | Yen-Ting Han(2021) | |  | [3] |
|  |  | Yen-Ting Han(2021) | |  | [3] |
|  | milling | R. Vinci(2020) | |  | [40] |
|  |  | R. Vinci(2020) | |  | [40] |
|  |  | R. Vinci(2020) | |  | [40] |
|  |  | R. Vinci(2020) | |  | [40] |

**Table S4.** Forest plot showing the horizontal apical deviations of the reviewed studies concerning different guide fabrication in different research types.

| Research type | Guide fabrication | Author(year) | Horizontal apical deviation (mm)  Mean±SD 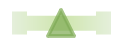Median (min,max) 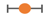 0 1 2 3 | Ref. |
| --- | --- | --- | --- | --- |
| *In vitro* | 3D printer | Chia-Cheng Lin(2020) |  | [29] |
|  |  | Chia-Cheng Lin(2020) |  | [29] |
|  |  | Chia-Cheng Lin(2020) |  | [29] |
|  |  | Kang-jie Cheng(2020) |  | [32] |
|  |  | Roberto Pessoa(2022) |  | [30] |
|  |  | Roberto Pessoa(2022) |  | [30] |
|  |  | Yao Sun(2022) |  | [21] |
|  |  | Yao Sun(2022) |  | [21] |
|  |  | David Schneider(2021) |  | [36] |
|  |  | David Schneider(2021) |  | [36] |
|  |  | Young Woo Song(2021) |  | [34] |
|  |  | Jaafar Abduo(2021) |  | [37] |
|  |  | Jaafar Abduo(2021) |  | [37] |
|  | milling | Young Woo Song(2021) |  | [34] |
|  |  | Jaafar Abduo(2021) |  | [37] |
|  |  | Jaafar Abduo(2021) |  | [37] |
| *In vivo* | 3D printer | Kristof Orban(2022) |  | [17] |
|  |  | Chalermchai Ngamprasertkit(2021) |  | [2] |
|  |  | Chalermchai Ngamprasertkit(2021) |  | [2] |
|  |  | Yen-Ting Han(2021) |  | [3] |
|  |  | Yen-Ting Han(2021) |  | [3] |
|  |  | Yen-Ting Han(2021) |  | [3] |
|  |  | Yen-Ting Han(2021) |  | [3] |
|  |  | Yen-Ting Han(2021) |  | [3] |
|  |  | Yen-Ting Han(2021) |  | [3] |
|  | milling | R. Vinci(2020) |  | [40] |
|  |  | R. Vinci(2020) |  | [40] |
|  |  | R. Vinci(2020) |  | [40] |
|  |  | R. Vinci(2020) |  | [40] |

# References

1. Lou F, Rao P, Zhang M, Luo S, Lu S, Xiao J. Accuracy evaluation of partially guided and fully guided templates applied to implant surgery of anterior teeth: A randomized controlled trial. Clin Implant Dent Relat Res. 2021;23(1):117-30.

2. Ngamprasertkit C, Aunmeungthong W, Khongkhunthian P. The implant position accuracy between using only surgical drill guide and surgical drill guide with implant guide in fully digital workflow: a randomized clinical trial. Oral Maxillofac Surg. 2022;26(2):229-37.

3. Han YT, Lin WC, Fan FY, Chen CL, Lin CC, Cheng HC. Comparison of Dental Surface Image Registration and Fiducial Marker Registration: An In Vivo Accuracy Study of Static Computer-Assisted Implant Surgery. J Clin Med. 2021;10(18).

4. Huang L, Zhang X, Mo A. A Retrospective Study on the Transferring Accuracy of a Fully Guided Digital Template in the Anterior Zone. Materials (Basel). 2021;14(16).

5. Chen Y, Zhang X, Wang M, Jiang Q, Mo A. Accuracy of Full-Guided and Half-Guided Surgical Templates in Anterior Immediate and Delayed Implantation: A Retrospective Study. Materials (Basel). 2020;14(1).

6. Wu D, Zhou L, Yang J, Zhang B, Lin Y, Chen J, et al. Accuracy of dynamic navigation compared to static surgical guide for dental implant placement. Int J Implant Dent. 2020;6(1):78.

7. Kniha K, Brandt M, Bock A, Modabber A, Prescher A, Holzle F, et al. Accuracy of fully guided orthodontic mini-implant placement evaluated by cone-beam computed tomography: a study involving human cadaver heads. Clin Oral Investig. 2021;25(3):1299-306.

8. Suksod N, Kunavisarut C, Kitisubkanchana J. Accuracy of computer-guided implantation in the placement of one-piece ceramic dental implants in the anterior region: A prospective clinical study. PLoS One. 2020;15(9):e0237229.

9. Skjerven H, Riis UH, Herlofsson BB, Ellingsen JE. In Vivo Accuracy of Implant Placement Using a Full Digital Planning Modality and Stereolithographic Guides. Int J Oral Maxillofac Implants. 2019;34(1):124-32.

10. Chen Z, Li J, Sinjab K, Mendonca G, Yu H, Wang HL. Accuracy of flapless immediate implant placement in anterior maxilla using computer-assisted versus freehand surgery: A cadaver study. Clin Oral Implants Res. 2018;29(12):1186-94.

11. Fang Y, An X, Jeong SM, Choi BH. Accuracy of computer-guided implant placement in anterior regions. J Prosthet Dent. 2019;121(5):836-42.

12. Ma B, Park T, Chun I, Yun K. The accuracy of a 3D printing surgical guide determined by CBCT and model analysis. J Adv Prosthodont. 2018;10(4):279-85.

13. Gjelvold B, Mahmood DJH, Wennerberg A. Accuracy of surgical guides from 2 different desktop 3D printers for computed tomography-guided surgery. J Prosthet Dent. 2019;121(3):498-503.

14. Feng Y, Su Z, Mo A, Yang X. Comparison of the accuracy of immediate implant placement using static and dynamic computer-assisted implant system in the esthetic zone of the maxilla: a prospective study. Int J Implant Dent. 2022;8(1):65.

15. Kivovics M, Penzes D, Nemeth O, Mijiritsky E. The Influence of Surgical Experience and Bone Density on the Accuracy of Static Computer-Assisted Implant Surgery in Edentulous Jaws Using a Mucosa-Supported Surgical Template with a Half-Guided Implant Placement Protocol-A Randomized Clinical Study. Materials (Basel). 2020;13(24).

16. Smitkarn P, Subbalekha K, Mattheos N, Pimkhaokham A. The accuracy of single-tooth implants placed using fully digital-guided surgery and freehand implant surgery. J Clin Periodontol. 2019;46(9):949-57.

17. Orban K, Varga E, Jr., Windisch P, Braunitzer G, Molnar B. Accuracy of half-guided implant placement with machine-driven or manual insertion: a prospective, randomized clinical study. Clin Oral Investig. 2022;26(1):1035-43.

18. Gargallo-Albiol J, Zilleruelo-Pozo MJ, Lucas-Taule E, Munoz-Penalver J, Paternostro-Betancourt D, Hernandez-Alfaro F. Accuracy of static fully guided implant placement in the posterior area of partially edentulous jaws: a cohort prospective study. Clin Oral Investig. 2022;26(3):2783-91.

19. Chang RJ, Chen HL, Huang LG, Wong YK. Accuracy of implant placement with a computer-aided fabricated surgical template with guided parallel pins: A pilot study. J Chin Med Assoc. 2018;81(11):970-6.

20. D'Haese R, Vrombaut T, Hommez G, De Bruyn H, Vandeweghe S. Accuracy of Guided Implant Surgery Using an Intraoral Scanner and Desktop 3D-Printed Tooth-Supported Guides. Int J Oral Maxillofac Implants. 2022;37(3):479-84.

21. Sun Y, Ding Q, Tang L, Zhang L, Sun Y, Xie Q. Accuracy of a chairside fused deposition modeling 3D-printed single-tooth surgical template for implant placement: An in vitro comparison with a light cured template. J Craniomaxillofac Surg. 2019;47(8):1216-21.

22. Herschdorfer L, Negreiros WM, Gallucci GO, Hamilton A. Comparison of the accuracy of implants placed with CAD-CAM surgical templates manufactured with various 3D printers: An in vitro study. J Prosthet Dent. 2021;125(6):905-10.

23. Guentsch A, Sukhtankar L, An H, Luepke PG. Precision and trueness of implant placement with and without static surgical guides: An in vitro study. J Prosthet Dent. 2021;126(3):398-404.

24. Sittikornpaiboon P, Arunjaroensuk S, Kaboosaya B, Subbalekha K, Mattheos N, Pimkhaokham A. Comparison of the accuracy of implant placement using different drilling systems for static computer-assisted implant surgery: A simulation-based experimental study. Clin Implant Dent Relat Res. 2021;23(4):635-43.

25. Henprasert P, Dawson DV, El-Kerdani T, Song X, Couso-Queiruga E, Holloway JA. Comparison of the Accuracy of Implant Position Using Surgical Guides Fabricated by Additive and Subtractive Techniques. J Prosthodont. 2020;29(6):534-41.

26. El Kholy K, Lazarin R, Janner SFM, Faerber K, Buser R, Buser D. Influence of surgical guide support and implant site location on accuracy of static Computer-Assisted Implant Surgery. Clin Oral Implants Res. 2019;30(11):1067-75.

27. D'Haese R, Vrombaut T, Hommez G, De Bruyn H, Vandeweghe S. Accuracy of Guided Implant Surgery in the Edentulous Jaw Using Desktop 3D-Printed Mucosal Supported Guides. J Clin Med. 2021;10(3).

28. Kauffmann P, Rau A, Engelke W, Troeltzsch M, Brockmeyer P, Dagmar LS, et al. Accuracy of Navigation-Guided Dental Implant Placement with Screw Versus Hand Template Fixation in the Edentulous Mandible. Int J Oral Maxillofac Implants. 2018;33(2):383-8.

29. Lin CC, Ishikawa M, Maida T, Cheng HC, Ou KL, Nezu T, et al. Stereolithographic Surgical Guide with a Combination of Tooth and Bone Support: Accuracy of Guided Implant Surgery in Distal Extension Situation. J Clin Med. 2020;9(3).

30. Pessoa R, Siqueira R, Li J, Saleh I, Meneghetti P, Bezerra F, et al. The Impact of Surgical Guide Fixation and Implant Location on Accuracy of Static Computer-Assisted Implant Surgery. J Prosthodont. 2022;31(2):155-64.

31. Baez-Marrero N, Rafel JL, Rodriguez-Cardenas YA, Aliaga-Del Castillo A, Dias-Da Silveira HL, Arriola-Guillen LE. Accuracy of computer-assisted surgery in immediate implant placement: An experimental study. J Indian Soc Periodontol. 2022;26(3):219-23.

32. Cheng KJ, Kan TS, Liu YF, Zhu WD, Zhu FD, Wang WB, et al. Accuracy of dental implant surgery with robotic position feedback and registration algorithm: An in-vitro study. Comput Biol Med. 2021;129:104153.

33. Singthong W, Serichetaphongse P, Chengprapakorn W. A randomized clinical trial on the accuracy of guided implant surgery between two implant-planning programs used by inexperienced operators. J Prosthet Dent. 2022.

34. Song YW, Kim J, Kim JH, Park JM, Jung UW, Cha JK. Accuracy of Dental Implant Placement by a Novel In-House Model-Free and Zero-Setup Fully Guided Surgical Template Made of a Light-Cured Composite Resin (VARO Guide((R))): A Comparative In Vitro Study. Materials (Basel). 2021;14(14).

35. Li-Rodriguez JK, Diaz-Durany M, Romeo-Rubio M, Paz Salido M, Pradies G. Accuracy of a guided implant system with milled surgical templates. J Oral Sci. 2022;64(2):145-50.

36. Schneider D, Sax C, Sancho-Puchades M, Hammerle CHF, Jung RE. Accuracy of computer-assisted, template-guided implant placement compared with conventional implant placement by hand-An in vitro study. Clin Oral Implants Res. 2021;32(9):1052-60.

37. Abduo J, Lau D. Accuracy of static computer-assisted implant placement in long span edentulous area by novice implant clinicians: A cross-sectional in vitro study comparing fully-guided, pilot-guided, and freehand implant placement protocols. Clin Implant Dent Relat Res. 2021;23(3):361-72.

38. Brandt J, Brenner M, Lauer HC, Brandt S. Accuracy of a Template-Guided Implant Surgery System with a CAD/CAM-Based Measurement Method: An In Vitro Study. Int J Oral Maxillofac Implants. 2018;33(2):328-34.

39. Spille J, Jin F, Behrens E, Acil Y, Lichtenstein J, Naujokat H, et al. Comparison of implant placement accuracy in two different preoperative digital workflows: navigated vs. pilot-drill-guided surgery. Int J Implant Dent. 2021;7(1):45.

40. Vinci R, Manacorda M, Abundo R, Lucchina AG, Scarano A, Crocetta C, et al. Accuracy of Edentulous Computer-Aided Implant Surgery as Compared to Virtual Planning: A Retrospective Multicenter Study. J Clin Med. 2020;9(3).
